# Giant peroxisomes in a moss (*Physcomitrella patens*) *peroxisomal biogenesis factor 11* mutant

**DOI:** 10.1111/nph.13739

**Published:** 2015-11-06

**Authors:** Yasuko Kamisugi, Shiro Mitsuya, Mahmoud El‐Shami, Celia D. Knight, Andrew C. Cuming, Alison Baker

**Affiliations:** ^1^ Centre for Plant SciencesFaculty of Biological SciencesUniversity of LeedsLeedsLS2 9JTUK

**Keywords:** gene disruption, organelle size, peroxisome, peroxisomal biogenesis factor 11 (PEX11), *Physcomitrella patens*

## Abstract

- Peroxisomal biogenesis factor 11 (PEX11) proteins are found in yeasts, mammals and plants, and play a role in peroxisome morphology and regulation of peroxisome division. The moss *Physcomitrella patens* has six PEX11 isoforms which fall into two subfamilies, similar to those found in monocots and dicots.
- We carried out targeted gene disruption of the *Phypa_PEX11‐1* gene and compared the morphological and cellular phenotypes of the wild‐type and mutant strains.
- The mutant grew more slowly and the development of gametophores was retarded. Mutant chloronemal filaments contained large cellular structures which excluded all other cellular organelles. Expression of fluorescent reporter proteins revealed that the mutant strain had greatly enlarged peroxisomes up to 10 μm in diameter. Expression of a vacuolar membrane marker confirmed that the enlarged structures were not vacuoles, or peroxisomes sequestered within vacuoles as a result of pexophagy. Phypa_PEX11 targeted to peroxisome membranes could rescue the knock out phenotype and interacted with Fission1 on the peroxisome membrane.
- Moss PEX11 functions in peroxisome division similar to PEX11 in other organisms but the mutant phenotype is more extreme and environmentally determined, making *P. patens* a powerful system in which to address mechanisms of peroxisome proliferation and division.

## Introduction

Peroxisomes are organelles found in all eukaryotic cells from unicellular eukaryotes such as algae and baker's yeast to complex multicellular organisms such as humans and flowering plants. Peroxisomes can be derived from the endoplasmic reticulum (ER) but can grow by post‐translational import of membrane and matrix proteins, divide and segregate into daughter cells (Fagarasanu *et al*., 2007) However, the relative importance of *de novo* biogenesis versus organelle division remains hotly debated (Hettema *et al*., 2014) and it should be noted that in plants no direct evidence for ER luminal connections has been found (Barton *et al*., 2013). Peroxisomes are also capable of proliferation under appropriate environmental conditions. In fungi, peroxisomes are induced to proliferate by nutritional cues, for example methanol in the case of methylotrophic fungi such as *Hansenula polymorpha* (van der Klei *et al*., 2006) or oleate in *Saccharomyces cerevisiae* (Gurvitz & Rottensteiner, 2006), and peroxisomes play essential roles in metabolism of these substrates. Peroxisomes are important sites of cellular defence against oxidative stress and several studies have reported peroxisome proliferation in response to stress conditions such as high light (Ferreira *et al*., 1989; Desai & Hu, 2008), salt (Palma *et al*., 1987; Mitsuya *et al*., 2010), heavy metals (Palma *et al*., 1987) and hydrogen peroxide (Lopez‐Huertas *et al*., 2000). Division and proliferation share common steps. Morphologically, peroxisomes first tubulate or elongate, followed by constriction, often giving a ‘beads‐on‐a string’ appearance, and finally divide (Thoms & Erdmann, 2005; Kaur & Hu, 2009).

In recent years, much has been learned about the molecular machinery of peroxisome proliferation and division (Schrader *et al*., 2012). The first identified component was *S. cerevisiae* peroxisomal biogenesis factor 11 (PEX11). Mutants disrupted in the *PEX11* gene have greatly enlarged peroxisomes, while cells overexpressing *PEX11* have large numbers of small peroxisomes (Erdmann & Blobel, 1995; Marshall *et al*., 1995). ScPEX11 is a peroxisome membrane protein the expression of which is strongly induced by oleate (Gurvitz *et al*., 2001), and the homologous protein from the fungus *Penicillium chrysogenum* has recently been shown to induce membrane curvature and tubulation of lipid vesicles *in vitro* (Opalinski *et al*., 2011). Fungal PEX11 has homologues in mammals and plants. In mammals there are three isoforms termed PEX11α, β and γ (Schrader *et al*., 1998; Li *et al*., 2002a). Pex11α is thought to have a role in peroxisome proliferation in response to external stimuli such as hypolipodaemic drugs and peroxisome proliferators, whereas Pex11β plays a role in constitutive proliferation/division (Schrader *et al*., 1998; Li *et al*., 2002b). The N‐terminal 40 amino acids of Pex11β, which include the second amphipathic helix, are also important for membrane tubulation *in vivo* (Bonekamp *et al*., 2013). Recently, the first patient with a mutation in Pex11β was described, who had enlarged and elongated peroxisomes and clinical symptoms similar to those of other patients with mild peroxisome disorders (Ebberink *et al*., 2012).

Plants have a still larger PEX11 family, with five genes in Arabidopsis (Lingard & Trelease, 2006) and rice (*Oryza sativa*; Nayidu *et al*., 2008) which fall into two clades, one containing Arabidopsis isoforms c, d and e and rice isoforms 1 and 2 and the other containing Arabidopsis isoforms a and b and rice isoforms 3, 4 and 5 (Orth *et al*., 2007). Phylogenetic analysis indicates that *PEX11* genes have a monophyletic origin and have evolved independently in the different kingdoms (Orth *et al*., 2007) (Chang *et al*., 2015) Evidence for (some) conservation of function comes from the ability of one Arabidopsis isoform (PEX11e) to partially complement the *S. cerevisiae pex11* mutant (Orth *et al*., 2007) and from the finding that human, yeast and plant PEX11 proteins have similar effects on peroxisome proliferation when expressed ectopically in human cells (Koch *et al*., 2010). Arabidopsis PEX11 b–e are proposed to span the membrane twice with both termini in the cytsol (Lingard & Trelease, 2006), and a similar topology was reported for mammalian Pex11β (Bonekamp *et al*., 2013).

Peroxisome division is achieved by a machinery that has shared components with mitochondria (Table 1) (Mano *et al*., 2004; Schrader, 2006; Zhang & Hu, 2008, 2009), and in plants with chloroplasts (Zhang & Hu, 2010). In current models, tail‐anchored proteins of the Fission1 (Fis1) family are targeted to peroxisomes as well as mitochondria and play a role in the recruitment of dynamin‐related proteins which sever the tubulated peroxisomes that result from binding of PEX11. PEX11 is the only peroxisome‐specific component of this machinery identified to date. Arabidopsis PEX11 proteins have been shown to both homo‐ and hetero‐oligomerize (Rahim *et al*., 2009) as well as interact with Fis1b (Lingard *et al*., 2008). In mammalian cells, homo‐oligomerization of PEX11β was observed, and a ternary complex containing Pex11β, Fis1 and dynamin like protein 1 could be identified (Kobayashi *et al*., 2007). In yeast, Pex11p also dimerizes in a redox‐sensitive manner (Marshall *et al*., 1996). ScPEX11 has been shown to be phosphorylated and activated by Pho85 kinase on Ser 165 and 167, phosphorylation promoting peroxisome association and proliferation (Knoblach & Rachubinski, 2010). In *Pichia pastoris*, phosphorylation of PEX11 Ser 173 promotes interaction with Fis1p and hyper‐divided peroxisomes but does not affect transit from the ER to peroxisomes (Joshi *et al*., 2012). Thus, PEX11 controls peroxisome proliferation in yeast through transcriptional (Gurvitz *et al*., 2001) and post‐transcriptional (Knoblach & Rachubinski, 2010) (Joshi *et al*., 2012) means. Other roles for PEX11 have been described. In *S. cerevisiae*, PEX11 plays a role in medium‐chain fatty acid oxidation (van Roermund *et al*., 2000) and has also been implicated in mitochondrial–peroxisome contact sites (Mattiazzi Usaj *et al*., 2015). In *Yarrowia lipolytica*,* pex11* mutants showed a peroxisome biogenesis defect; they lacked peroxisomes and mislocalized matrix proteins to the cytosol (Chang *et al*., 2015).

**Table 1:** Peroxisome division components in humans, yeast, Arabidopsis and moss

	*Homo sapiens*	*Saccharomyces cerevisiae*	*Arabidopsis thaliana*	*Physcomitrella patens*
Peroxisome tubulation	PEX11α PEX11β PEX11γ	PEX11 PEX25 PEX27	PEX11a PEX11b	Phypa_439417 (Phypa_PEX11‐5) Phypa_447344 (Phypa_PEX11‐3) Phypa_428462^a^ (Phypa_PEX11‐6) Phypa_426510 (Phypa_PEX11‐4) Phypa_422942^a^
			PEX11c PEX11d PEX11e	(Phypa_PEX11‐2) Phypa_460481 (Phypa_PEX11‐1)
DRP tether	hFIS1 Mff?	Fis1p	FIS1A FIS1B?	Phypa_433535
Soluble adaptors		Caf4p Mdv1p		Phypa_436928 (annotated as mt fission factors)
Dynamin‐like/related proteins (fission)	DRP1	Dmn1p Vps1p	DRP3A DRP3B	Phypa_433610 Phypa_449578 Phypa_452114 Phypa_4389781
			DRP5B	Phypa_431552 Phypa_456622 Phypa_454673

a V1.6 gene models are incorrect.

*Physcomitrella patens* is an excellent model for comparative plant cell biology. It belongs to the bryophytes, the first group to diverge from the plant lineage following the conquest of land, and was the first nonflowering plant to have its genome sequenced, facilitating comparative genetic analysis (Rensing *et al*., 2008). Unlike flowering plants, *P. patens* exhibits somatic homologous recombination at high frequency, facilitating the production of knockout mutant lines where the precise site of transgene integration can be controlled (Schaefer & Zryd, 1997). Although *P. patens* has been reported to contain peroxisomes of the glyoxysome type (Huang *et al*., 2009) and the distribution and quantification of peroxisomes have been reported (Furt *et al*., 2012), no further cellular or molecular characterization of moss peroxisomes has been described. Here, we describe the PEX11 family in *P. patens* and the phenotype of a knockout mutant in one member of the gene family which results in the formation of giant peroxisomes.

## Materials and Methods

### Identification of *Phypa_PEX11* genes

Database searching with the Arabidopsis *PEX11e* gene identified the cDNA clone PPN181002 (Accession BI436924) as a potential *Physcomitrella PEX11* orthologue. Within the *P. patens* genome assembly, version 1.1, this corresponds to Protein ID Phypa1_1:200510. Additional *P. patens* gene models encoding *PEX11* homologues were identified by BLASTP search using Phypa1_1:200510 and the Arabidopsis PEX11 polypeptide sequences. One further gene model was identified with high similarity to Phypa1_1:200510 and the AtPEX11c and AtPEX11d sequences, and four further models with similarity to the AtPEX11a and AtPEX11b sequences (Table 1; Supporting Information Table S1).

### Plant material

The ‘Gransden’ strain of *Physcomitrella patens* (Hedw.) B.S.G. was propagated as a protonemal culture on BCD agar medium containing 1 mM CaCl_2_ and 5 mM ammonium tartrate (BCDAT), overlaid with cellophane, and as individual plants (‘spot inocula’) on the same medium without cellophane overlay. Protonemal tissue was vegetatively propagated by homogenization and subcultured every 7 d (Knight *et al*., 2002). For growth testing, small protonemal explants were inoculated onto BCD or BCDAT agar and plant growth was monitored during a 31‐d period following inoculation. The extent of plant growth was estimated following digital photography of the plates (Kamisugi *et al*., 2012). The image analysis software imagej (Abramoff *et al*., 2004) was used to convert the digital images to binary format and determine the colony area based on counting the number of pixels corresponding to each colony. Colony area determinations based on different photographs were normalized for each colony using the estimated area of the plate.

### RNA isolation and cDNA sequence analysis

A polysomal fraction was isolated from 7‐d‐old chloronemal tissue. Chloronemata were harvested from cellophane overlays, residual liquid was squeezed out between two sheets of filter paper and *c*. 0.3 g was ground to a powder then homogenized in 25 ml of 200 mM sucrose, 50 mM Tris‐Cl, pH8.5, 60 mM KCl, 30 mM MgCl_2_ and 1% (v/v) Triton X‐100. Following centrifugation at 25 000 ***g*** for 20 min, the supernatant was layered over a 5‐ml sucrose cushion (1 M sucrose, 40 mM Tris‐Cl, pH8.5, 20 mM KCl and 10 mM MgCl_2_) and centrifuged at 141 000 ***g*** for 3 h. The supernatant was aspirated and the pellet drained, for RNA extraction with 0.5 ml of extraction buffer (Knight *et al*., 2002). For rt‐PCR, RNA (*c*. 100 μg) was digested with 1 unit of RQ1 DNase (Promega) for 10 min at room temperature and purified by phenol‐chloroform extraction and ethanol precipitation. Complementary DNA was synthesized from 1 μg of RNA using the Promega Reverse Transcription System. The reaction mixture was diluted 5‐fold with water, and 2‐μl aliquots were used for PCR amplification. Primers used are listed in Table S2. The PCR products were cloned into pBluescript II KS^−^ and sequenced using T7/T3 universal primers.

### Plasmid constructions

#### Construction of the knock out vector pJHB1

**Figure 1:**
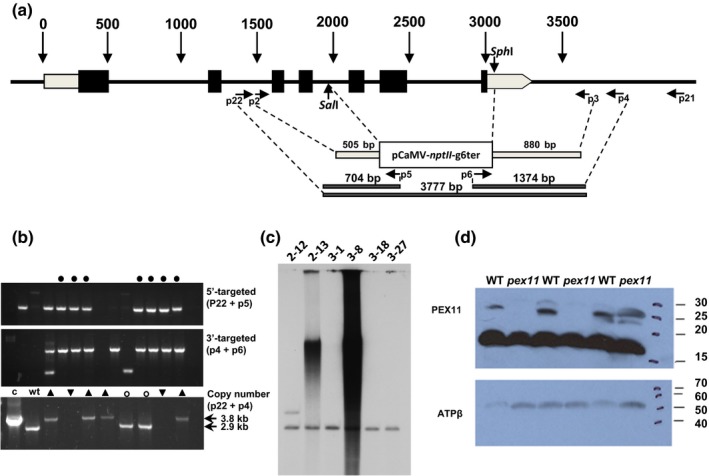
Targeted knockout of the *Physcomitrella patens Phypa_PEX11‐1* gene (*PEX11: peroxisomal biogenesis factor 11*). (a) Structure of the *Phypa_PEX11‐1* gene and design of the targeting construct. Exons are denoted by boxes (grey, untranslated region (UTR); black, protein‐coding region). The gene targeting construct comprised 505‐bp and 880‐bp 5′‐ and 3′‐targeting sequences amplified from the *Phypa_PEX11‐1* gene, and interrupted by replacing the sequences between the *Sph*I and *Sal*I sites with the *nptII* selection cassette. Dashed lines indicate the relationship of PCR primer pairs p2 and p3, p22 and p5, p6 and p4 and p22 to the sequence, and the fragments amplified from targeted transgenic strains using these primer combinations are indicated. (b) Identification of targeted strains by PCR. Targeting by the 5′ end of the targeting construct is indicated by the amplification of a 677‐bp fragment with primers p22 and p5 (top panel); targeting by the 3′ end of the construct by amplification of a 1374‐bp fragment using primers p4 and p6 (middle panel). Strains in which targeting at both ends has occurred are indicated by a black dot above the cognate tracks. The bottom panel identifies strains in which targeted replacement of the native locus with a single copy of the targeting construct has occurred, by amplification of a 3777‐bp fragment with primers p22 and p4. Track ‘c’ shows the amplification of the expected fragment in a control amplification of the disrupted, cloned gene from plasmid DNA. ‘wt’ indicates amplification of the native gene (2.9 kb) in a wild‐type (WT) strain. Lines indicated by upward arrowheads have undergone single‐copy targeted gene replacement. Lines indicated by downward arrowheads contain a multicopy replacement of the endogenous gene (too large for amplification by external primers) and lines indicated by open circles contain multicopy replacements of the endogenous locus but also a wild‐type copy of the native gene: these lines are polyploids formed by protoplast fusion during PEG‐mediated transformation. (c) Identification of strains lacking adventitious transgene insertion by Southern blot hybridization using the selection cassette sequence as a probe. Lines 2‐13 and 3‐8 are lines in which multiple copies of the transforming DNA have inserted at nontargeted sites within the genome. Line 2‐12 appears to contain a single off‐target insertion in addition to a correctly targeted locus and lines 3‐1, 3‐18 and 3‐27 contain only a single copy of the targeting cassette correctly targeted to the *Phypa_PEX11‐1* locus. (d) Western blot of wild‐type and three targeted mutants. Upper panel, probed with anti‐PEX11 antibody; lower panel, the same samples probed with anti‐ATPβ as a loading control. Samples 1 and 2 derive from targeted replacement lines. Sample 3 derives from a line containing a targeted insertion at the *PEX11‐1* locus, but retains a wild‐type copy of the gene. The intense band at *c*. 17 kDa in the upper panel is an unrelated cross‐reactive protein.

The cDNA clone PPN181002 was used to identify a BAC clone (pMOKM102M14) by screening a *Physcomitrella* BAC library. A 4.7‐kb *Eco*RI fragment containing the 3′‐terminal region of the *Phypa_PEX11* gene (containing the last five exons in the coding sequence) was subcloned and a 3.65‐kb fragment amplified by PCR using primers P22 and P21 (Fig. 1; Table S2) and subcloned into pDONR 201 to make the plasmid pJOB1. This plasmid was digested with *Sal*I and *Sph*I and re‐ligated with the *Sph*I/*Xho*I excised *nptII* cassette (p35S‐*nptII*‐CaMV g6ter) from the plasmid pMBL5 (Knight *et al*., 2002; Accession DQ228130) replacing the three C‐terminal protein‐coding exons. The resulting plasmid was recombined with pMBL6attR (Kamisugi *et al*. 2005) (Accession DQ228132), to generate the gene disruption construct pJHB1 (Fig. S1a). A markerless *PEX11* deletion construct was created by digesting pJHB1 with *Nco*I and *Bsp120*I to remove the selection cassette and re‐ligating the plasmid.

#### Construction of GFP and RFP reporters

GFP and RFP reporter constructs targeted to peroxisomes by C‐terminal addition of the peroxisome targeting signal 1 tripeptide ‘SKL’ (GFP and CFP (Sparkes *et al*., 2005)) or ‘SRL’ (RFP) were used to visualize gene expression in regenerated moss protonemata, for stable (RFP), unstable (GFP) and transient (CFP) expression. The plasmid pAmRFP‐SRL‐H108 was created by inserting a rice *actin1* promoter from pAct‐p and the mRFP‐SRL‐35Ster fragments from p35S::mRFP‐SRL (Pracharoenwattana *et al*., 2005) into the Acc65I site of pMBLH108 (Fig. S1b) to allow targeting of the reporter gene to the neutral ‘λ108’ locus (Schaefer & Zryd, 1997) To generate *PEX11* overexpression lines, cDNA of *PhypaPEX11‐1* was amplified with a pair of gene‐specific GATEWAY primers (Table S2) and cloned into pDONR 201 to create the entry vector pcPex11GW. The PEX11 fragment was subsequently recombined into a λ108‐targeting GFP‐fusion destination vector, pS65T‐GW‐H108, to create the overexpression reporter plasmid, pS65T‐Pex11GW (Fig. S1c).

### Moss transformations and molecular characterization

#### Gene disruption lines

Moss disruption lines for *Phypa_PEX11‐1* were generated by gene targeting, using a PCR‐amplified sequence from pJHB1. Primers Phypa_PEX11KOF and Phypa_PEX11KOR (Table S2; primers ‘p2’ and ‘p3’ in Fig. 1a) amplified a fragment of 3390 bp, comprising the p35S‐*nptII‐*CaMV g6ter cassette flanked by 505 bp of the *Phypa_PEX11* gene immediately 5′ to the *Sal*I site and 880 bp immediately 3′ to the *Sph*I site. Protoplasts were transformed using 15 μg of PCR fragment, and stable transformants were regenerated by three successive cycles of subculture on selective (containing G418 at 30 μg ml^−1^ (Melford Laboratories, Suffolk, UK)) and nonselective media as described previously (Knight *et al*., 2002; Kamisugi *et al*., 2005). Stable transformants were analysed by PCR to identify lines containing single‐copy targeted gene replacements, using external gene‐specific primers in conjunction with selection‐cassette‐specific primers as described by (Kamisugi *et al*., 2006) to identify targeting at the 5′ (primer pair p22 and p5 in Fig. 1a,b) and 3′ ends (primer pair p4 and p6 in Fig. 1a,b), and the external primers (p22 and p4) to distinguish between transformants in which the *Phypa_PEX11‐1* gene was disrupted by a single or multiple copies of the replacement cassette. Southern blotting was undertaken to identify plants in which no transforming DNA had adventitiously inserted at ectopic sites (Fig. 1c). Eight transgenic lines containing a targeted gene replacement, with no adventitious transgene copies, were obtained.

#### Transgenic reporter lines

Protoplasts were transformed with reporter constructs and transformants were regenerated, selected and maintained on medium containing 50 μg ml^−1^ hygromycin, as described above. To generate *Phypa_pex11‐1‐KO* mutants in a transgenic moss strain (G418^R^) expressing a GFP‐AtVam3 (Arabidopsis homologue of *S. cerevisiae* VAM3) fusion protein (Oda *et al*., 2009), this strain was transformed with a DNA fragment produced by PCR (using primers p2 and p3) from a derivative of pJHB1 from which the antibiotic selection cassette had been deleted. This *PEX11‐1* markerless fragment was co‐delivered to the GFP‐AtVam3 line with pAmRFP‐SRL‐H108 by protoplast‐PEG mediated transformation. The transformants were selected for hygromycin resistance to identify mRFP‐transformed plants and then by inspection for the *pex11‐1* knockout phenotype described in the Results. Correctly targeted deletion of the *PEX11‐1* gene in phenotypically identified mutants was confirmed by PCR using the external gene‐specific primers p22 and p4. To generate pex11‐GFP overexpression lines, 15 μg of pS65T‐Pex11GW linearized with *Swa*I was used to transform protoplasts as described above.

#### Transient expression by microprojectile bombardment

For bimolecular fluorescence complementation (BiFC) experiments, cDNA of *Phypa_PEX11‐1* and *PhypaFis1a/b* was amplified by PCR (primers listed in Table S2) and cloned into plasmids containing the N‐ and C‐terminal halves of YFP (pYFPn‐GS and pYFPc‐GS, respectively) to create pYFPn‐PEX11‐1 (Fig. S1d) and pYFPc‐Fis1a/b (Fig. S1e).

Equal quantities of each pair of YFP constructs and a transformation marker construct (pCFP‐SKL) were mixed and delivered to both wild‐type and mutant protonemal tissue by microprojectile bombardment, using a PDS1000 Biolistic system (Bio‐Rad, Hemel Hempstead, UK). Plasmid DNA (0.7–1 μg per shot) was bound to tungsten microprojectiles (M17; Bio‐Rad) (Sanford *et al*., 1993) for delivery using a 900‐psi rupture disc and a distance of 6 cm from the stopping screen. Following bombardment, plant tissue was incubated for 24–48 h at 25°C, before microscopic examination.

### Antibodies and western blotting

A polyclonal antiserum was raised in rabbit to the peptide VLYLNKAEARDKICRAIQYGSKFLSC corresponding to amino acids 15–40 of Arabidopsis PEX11e and affinity purified (Mitsuya *et al*., 2010). This sequence is specific to the PEX11c/d/e/clade (Figs S2, S3). Moss filaments were scraped from the cellophane‐overlaid agar plates after squeezing out residual liquid, and ground to a fine powder in liquid nitrogen in a mortar and pestle. Homogenization buffer (50 mM Tris HCl, pH8.2, 2 mM EDTA, 20% w/v glycerol, 1 mM PMSF, 2% complete protease inhibitor cocktail (Roche) and 0.5 mM DTT) was added at a ratio of 1 ml g^−1^ fresh weight of sample and ground to a fine slurry. The homogenate was centrifuged at 800 ***g*** for 10 min at 4°C and the supernatant was centrifuged at 164 000 ***g*** for 30 min at 6°C. The supernatant was removed and the pellet (crude membranes) was resuspended in 30 μl of homogenization buffer plus 5 μl of 10% w/v SDS. The protein concentration was measured using the BCA assay (Pierce, https://www.thermofisher.com/order/catalog/product/23225). Western blotting was as described previously (Mitsuya *et al*., 2010).

### Fluorescence microscopy

Microscopic imaging of moss cells was undertaken using a Zeiss AxioImager M2 microscope with Nomarski optics and an HXP120C light source for fluorescence imaging. Zeiss filter cubes with Semrock narrow‐band pass filters were used for CFP (CFP‐2432C), YFP (46 HE) GFP (38 HE) and RFP (TRITC‐B) detection. Images were captured with an Axiocam MRM camera and processed using Axiovision software. An LSM510 META confocal microscope (Carl Zeiss Ltd, Hertfordshire, UK) with ×40 Plan Neofluar (oil‐immersion; N.A. = 1.3) objective was used for confocal imaging. Argon laser excitation (wavelength: 488 nm) was used to excite GFP‐SKL. The fluorescent signal was detected though a 500–550‐nm band pass filter using the single track function (Sparkes *et al*., 2005). For co‐expression imaging of GFP and RFP, argon (488 nm) and neon (543 nm) lasers, respectively, were used for excitation with alternate line switching of multitrack function. The fluorescent signal was detected through a 500–530‐nm band pass filter for GFP and a 565–615‐nm band‐pass filter for RFP following the 545‐nm dichroic beam splitter.

## Results

### The *Physcomitrella patens PEX11* gene family

A search of the ‘version 1’ *P. patens* genome assembly identified six potential *Phypa_PEX11* gene sequences, which we designated *Phypa_PEX11‐1* to *Phypa_PEX11‐6*. The two sequences most similar to the *AtPEX11cde* clade comprised *Phypa_PEX11‐1* and *Phypa_PEX11‐2*, and the four most similar to the *ab* clade were designated *Phypa_PEX11‐3*,* 4*,* 5* and *6*, respectively (Table 1). Close inspection of V1.1 ‘filtered models’ suggested that the predicted polypeptide sequences of these were incorrect, but that other models were well supported by cDNA evidence. Subsequent versions of the genome assembly and annotations have corrected these models. A summary of gene identifiers in successive genome annotations is provided in Table S1, while Table 1 includes the gene and protein IDs according to the current V1.6 annotation. The model for *Phypa_PEX11‐2* lacked cDNA support, but sequence similarity between *Pp‐PEX11‐1* and *Phypa_PEX11‐2* suggested that the exon 2/intron 3 splice junction was mis‐specified in the latter, and that the C‐terminal sequence was incorrect because of the mis‐specification of exon 6, and a failure to identify a seventh exon. Of the four polypeptides similar to AtPEX11a and AtPEX11b (Phypa1_1:80254/Pp1s84_2898V6, Phypa1_1:62335/Pp1s16_338V6, Phypa1_1:63102/Pp1s159_21V6 and Phypa1_1:118714/Pp1s25_244V6), the first three were supported by cDNA sequences. The fourth lacked experimental support (Table 1).

In order to resolve the two unsupported models, rt‐PCR was conducted using primers specific for the hypothetical 5′ and 3′ untranslated region (UTR) sequences of *Phypa_PEX11‐2* and *Phypa_PEX11‐6* to amplify the corresponding sequences from mRNA obtained from a polyribosomal fraction (in order to avoid the amplification of possible splicing intermediates). In each case a transcript was amplified, and sequence analysis of these enabled the correct structure of the two genes to be confirmed. For the *Phypa_PEX11‐2* gene, splicing of intron 3 makes use of a 5′‐GC splice junction. While noncanonical, such splice junctions are not unusual, and have been observed for other *P. patens* genes. The sequence analysis additionally confirmed the exon 6–intron 6–exon 7 structure predicted by sequence homology. This sequence was deposited in GenBank as accession JQ026023, and the model has only recently been correctly assigned in the most recent annotation of the *P. patens* genome (as Pp3c18_11990V1 in the ‘version 3.1′ genome assembly prerelease (https://www.cosmoss.org/fgb2/gbrowse/V3.1/)). For *Phypa_PEX11‐6*, cDNA sequencing confirmed the accuracy of the models in the two V3 assemblies. We constructed a multiple sequence alignment of PEX11 sequences identified in a range of plant genomes. All show extensive homology throughout the conserved PEX11 domain (pfam05648) (Figs S2, S3) especially in the N‐terminal third of the protein which includes the amphipathic helix implicated in membrane tubulation (Fig. S4; Opalinski *et al*., 2011).

### Phypa_PEX11‐1 knockout mutants show developmental delay

The relative abundance of expressed sequence tags within the *P. patens* sequence database indicates the most highly expressed member of the gene family to be the PEX11cde member that we designated *Phypa_PEX11‐1*. Additionally, results of a digital gene expression (RNA‐seq) analysis of the chloronemal transcriptome indicate this gene to be expressed at a level *c*. 4000‐fold higher than *Phypa_PEX11‐2* and between 11 and 500 times higher than the *Phypa_PEX11ab*‐class transcripts (Table S3; Whitaker *et al*., 2010). The *Phypa_PEX11‐1* gene was disrupted by the targeted replacement of its last three exons by an *nptII* selection cassette (Fig. 1a). Lines containing single‐copy disruption alleles were identified by PCR amplification of the targeted locus (Fig. 1b), and the absence of adventitious genomic incorporation of the targeting construct was verified by Southern blot hybridization (Fig 1c). Eight genetically identical ‘clean’ disruption lines were identified. Western blot analysis with an antibody specific for the PEX11cde clade of two lines confirmed as containing a disrupted *Phypa_PEX11‐1* gene demonstrated the lack of accumulation of a PEX11 polypeptide in these knockout lines: a third line identified as retaining a wild‐type copy, and derived from a targeted insertion event (Kamisugi *et al*., 2006) rather than a targeted replacement, showed normal accumulation of PEX11 (Fig. 1d). Subsequent characterization was performed on line 3–18 (Fig. 1c). As *P. patens* performs high‐efficiency homologous recombination, all lines shown by PCR and Southern blot to be correctly targeted and to contain only a single copy of the transgene are genetically identical: this line is representative of all such lines.

**Figure 2:**
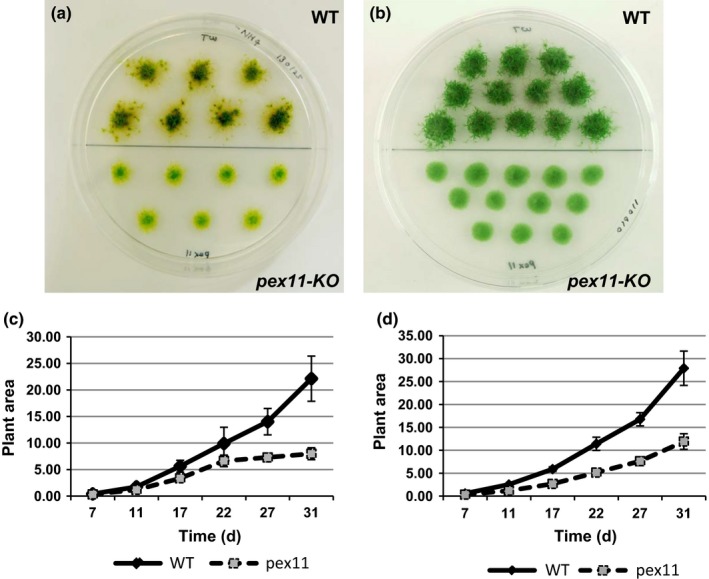
Growth of wild‐type (WT) and *pex11‐1* mutants. (a) Appearance of wild‐type (upper 7) *Physcomitrella patens* plants and *Phypa_pex11‐1‐KO* (lower 7) plants grown on BCD agar medium for 31 d following inoculation with protonemal explants. A dark‐green central zone (principally chloronemata) is surrounded by a pale‐green diffuse network of caulonemal filaments, from which gametophores can be seen developing in profusion in the wild‐type strain. Gametophore differentiation is comparatively retarded in the *pex11KO* mutant. (b) Appearance of wild‐type (upper 12) plants and *Phypa_pex11‐1‐KO* (lower 12) plants grown on BCD containing 1 mM CaCl_2_ and 5 mM ammonium tartrate (BCDAT) for 31 d following inoculation with protonemal explants. Supplementation with ammonium tartrate favours chloronemal development. While the wild‐type plants have developed a large number of gametophores, the mutant plants remain entirely protonemal in character on BCDAT medium. (c) Growth rate of wild‐type (solid line) and mutant (dashed line) plants on BCD agar medium. Growth is measured by determining the surface area of each plant (mean ± SD). (d) Growth rate of wild‐type (solid line) and mutant (dashed line) plants on BCDAT agar medium. Growth is measured by determining the surface area of each plant (mean ± SD) (arbitrary units).

**Figure 3:**
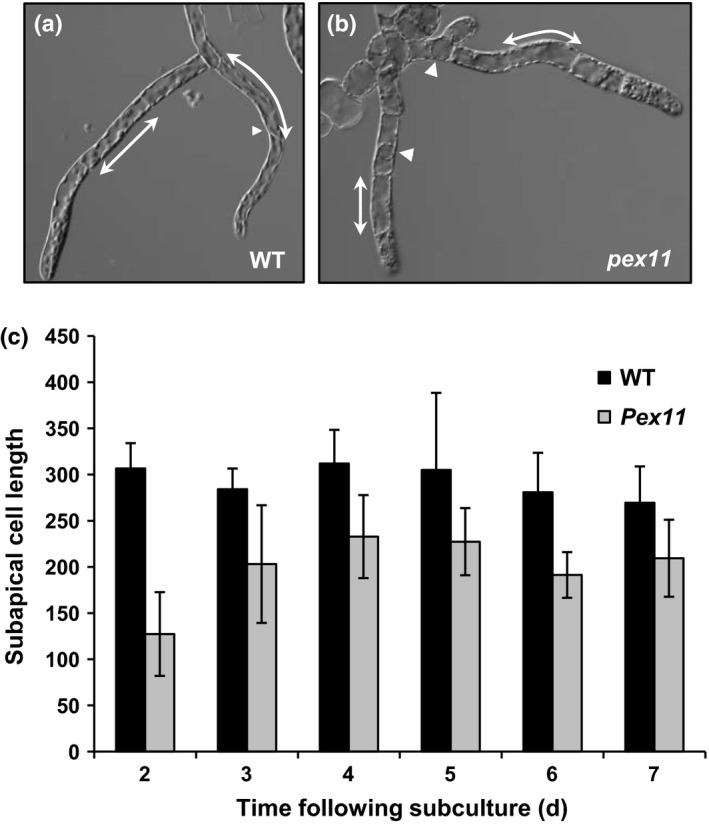
Reduced growth of the mutant is attributable to reduced cell elongation. (a) Regenerating protonemata of wild‐type *Physcomitrella patens* 4 d following fragmentation. The subapical cells measured in the determination of cell growth are indicated by the arrowed lines. While the branch to the left is chloronemal in nature, the main filament has already commenced differentiation into a caulonema, as evidenced by the oblique cross‐wall between the apical and subapical cells (arrowhead), the more sharply pointed apical dome, and the reduced number of chloroplasts. (b) Regenerating protonemata of the *pex11* mutant, 4 d following fragmentation. The subapical cells measured in the determination of cell growth are indicated by the arrowed lines. Large intracellular globular structures are evident in these cells (arrowheads). (c) Subapical cell lengths of regenerating protonemata measured at 2–7 d following fragmentation. Between 25 and 35 cells were measured at each time‐point. Average subapical cell length (arbitrary units) were measured in the wild type varies from 240% greater (2 d) to 128% greater (7 d) than in the *pex11* mutant. Error bars represent ± SD (*n *= 12–24).

The growth parameters of the *Phypa_PEX11‐1* mutant strains were compared with those of the wild‐type. The growth of mutant plants was significantly slower than that of the wild‐type strain, as determined by growth rate measurements. Additionally, the developmental transition from a primary filamentous stage (‘juvenile’) to the development of gametophores (‘adult’) was retarded in the mutant lines (Fig. 2). We ascribe these differences to reduced cellular elongation, and a retarded transition between chloronemal and caulonemal development in the *pex11‐1* mutant. First, the size of plants developing from small tissue explants on agar medium (‘spot inocula’) was significantly reduced both on medium containing nitrate as the sole nitrogen source (Fig. 2a,c) and on medium supplemented with ammonium tartrate, which favours chloronemal growth over caulonemal growth (Fig. 2b,d). The reduced differentiation of gametophores is particularly apparent in the mutant plants growing on nitrate medium (Fig. 2a). The parameters of cell elongation were determined by preparing homogenates of the wild‐type and mutant plants, in order to initiate tissue regeneration from small fragments of chloronemal tissue dispersed on cellophane‐overlaid agar medium. The terminal intact cell in each such fragment becomes re‐programmed as an apical stem cell. This cell undergoes repeated mitosis and cell elongation, to extend the length of the filament. Although the subapical cell will undergo mitosis to generate side‐branches, it does not elongate further. Thus, the lengths of the subapical cells provide a measure of the extent of cell elongation in each filament. When the lengths of the subapical cells of multicellular filaments were measured over a period of 1 wk following fragmentation, it was clear that the subapical cell length was significantly greater in the wild‐type filaments (Fig. 3). Additionally, caulonemal development could be identified in regenerating wild‐type tissue fragments at an earlier time than in the mutant (Fig. 3a). We also sometimes observed a rather swollen cell morphology in the regenerating mutants (Fig. 3b). When gametophores eventually developed, their appearance was phenotypically indistinguishable from that of gametophores borne by the wild‐type strain. Sporophyte development also appeared normal, and the spores produced were viable and germinated at a rate comparable to that of wild‐type spores (data not shown).

### Enlarged peroxisomes in the *Phypa_pex11‐1‐KO* mutant

**Figure 4:**
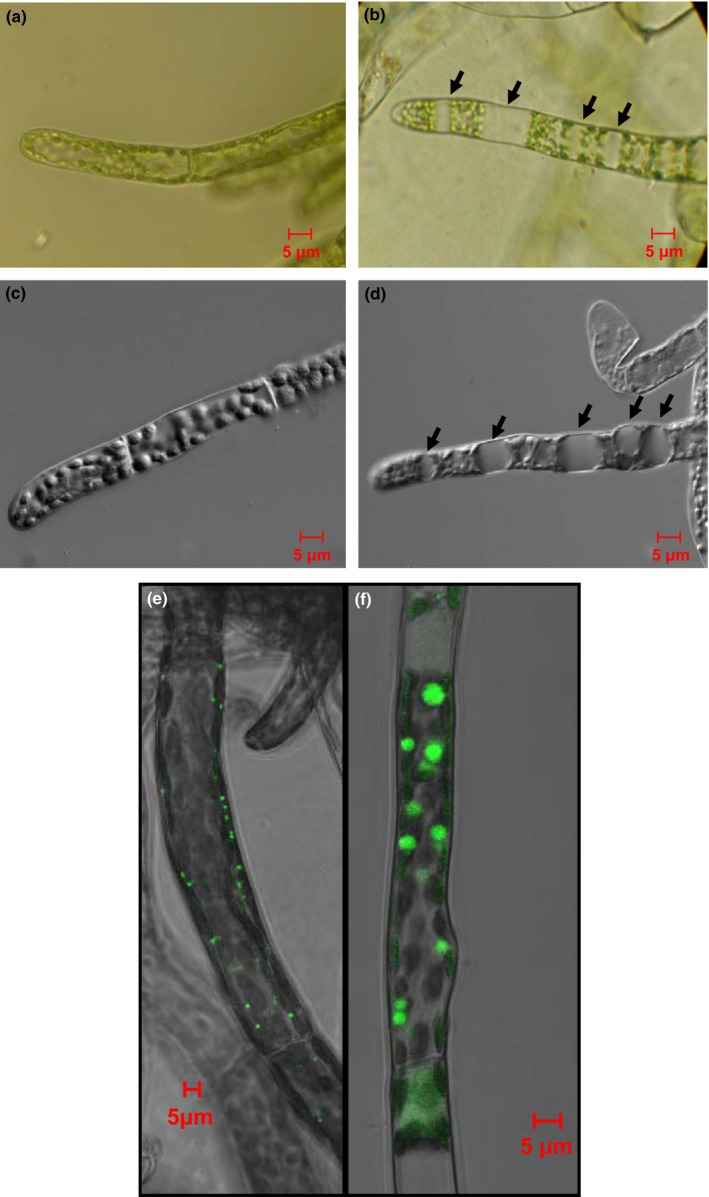
*Physcomitrella patens pex11‐1‐KO* mutants accumulate giant peroxisomes. (a–d) Bright‐field images of wild‐type (a, c) and *Phypa_pex11‐1‐KO* (b, d) protonemal cells visualized using standard (a, b) and Nomarski (c, d) optics. Giant peroxisomes are indicated by arrows in (b) and (d). (e, f) Confocal imaging of accumulation of the peroxisomal marker GFP‐SKL in wild‐type and mutant protonemata. (e) Wild type; (f) *Phypa_pex11‐1‐KO* mutant.

**Figure 5:**
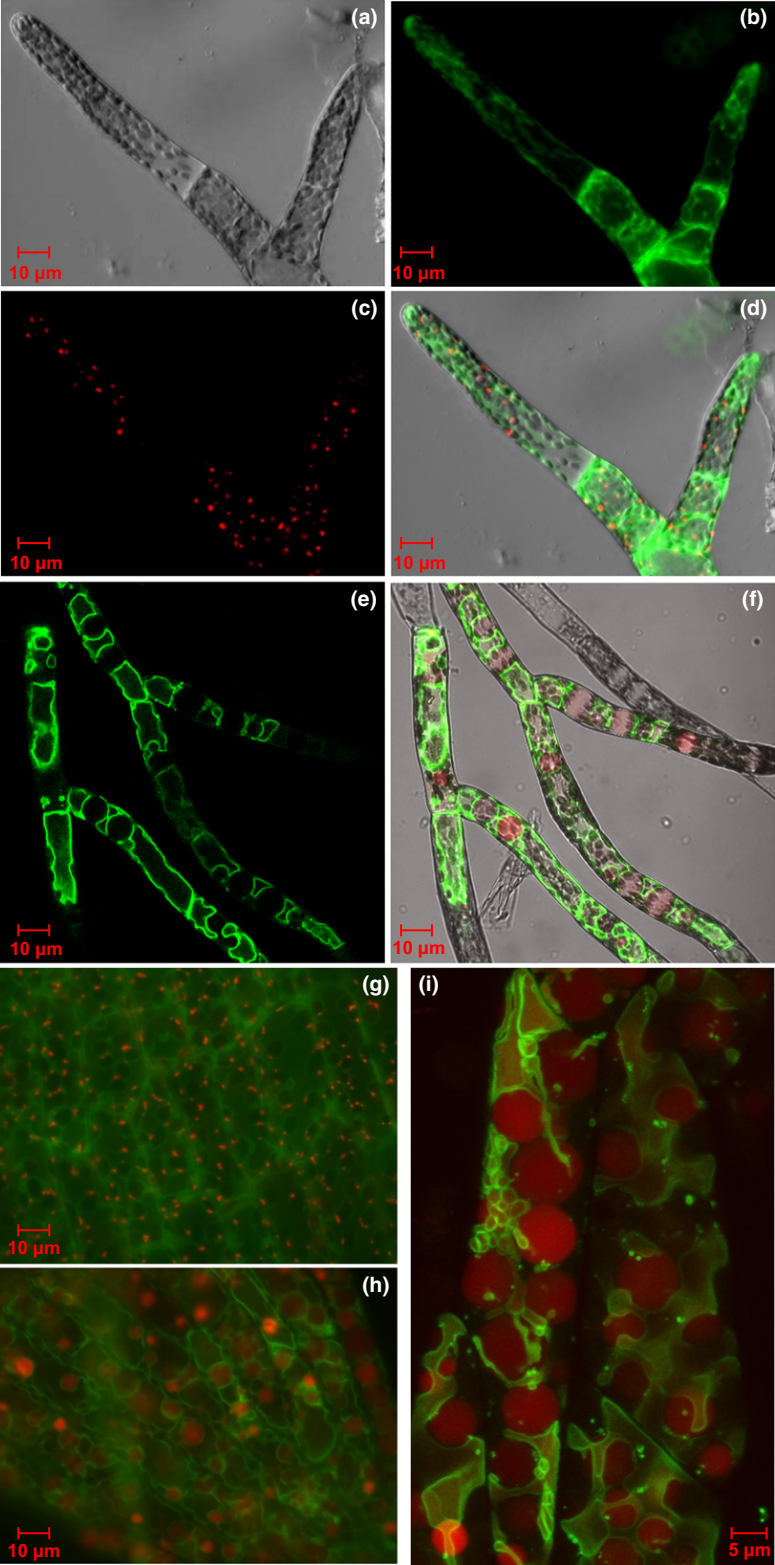
The giant organelles are peroxisomes not vacuoles. (a–d) Confocal images of vacuoles and peroxisomes in wild‐type protonemata of *Physcomitrella patens* transformed with the vacuolar reporter Arabidopsis homologue of *S. cerevisiae* VAM3 (AtVAM3)‐GFP and the peroxisome marker mRFP‐SRL. (a) Bright‐field image; (b) GFP fluorescence; (c) RFP fluorescence; (d) merged image. (e, f) Confocal images of vacuoles and peroxisomes in protonemata of the *Phypa_pex11‐1‐KO* mutant. (e) AtVAM‐GFP decorates the vacuolar membrane; (f) merged image for AtVAM‐GFP and mRFP‐SRL shows the peroxisomal marker filling large structures that distort the vacuolar membrane around them. (g, h) Epifluorescence microscopic images of vacuoles and peroxisomes in gametophore tissue of wild type (g) and *pex11* mutant (h) transformed with the vacuolar reporter AtVAM3‐GFP and the peroxisome marker mRFP‐SRL: merged fluorescent and bright‐field images. (i) Confocal z stack image showing giant peroxisomes (red) and vacuolar membrane (green) in gametophore cells of the *Phypa_pex11‐1‐KO* mutant transformed with the vacuolar reporter AtVAM3‐GFP and the peroxisome marker mRFP‐SRL: merged GFP and RFP images.

Inspection of protonemal filaments in the knockout lines revealed unusual structures. The cellular contents were distorted by the presence of very large, apparently empty globular structures (Fig. 4b,d) which were not present in the wild‐type (Fig. 4a,c). Mutation of the *AtPEX11* genes results in aberrant peroxisome division, and the formation of larger peroxisomes (Lingard & Trelease, 2006; Orth *et al*., 2007), but these do not approach the size of those we observed in *P. patens*. To determine whether these unusual structures in the moss mutants might correspond to massively enlarged peroxisomes or were simply vacuolar compartments, we investigated the localization of vacuolar and peroxisomal reporters. First, transient expression of a GFP reporter containing a peroxisomal targeting sequence (GFP‐SKL) in transgenic plants with either a wild‐type or a *Phypa_pex11‐1 KO* background supported this observation, with the mutant strain accumulating fewer, larger GFP‐labelled organelles (Fig. 4e,f). Next, in order to distinguish between peroxisomal membranes and vacuolar membranes, we additionally generated *Phypa_pex11‐KO* lines in a transgenic strain of *P. patens* containing an AtVAM3‐GFP reporter, previously shown to reveal tonoplast membranes in protonemata (Oda *et al*., 2009). These lines were generated using a markerless *Phypa_PEX11‐1* deletion construct co‐transformed with a plasmid containing a hygromycin resistance gene and an mRFP‐SRL reporter construct, so that the peroxisomes could be distinguished from the vacuoles. Peroxisomes and vacuoles were clearly distinguished in the wild type (Fig. 5a–d). *Phypa_pex11‐1Δ* lines were identified by their characteristic phenotype and those strains retaining a copy of the mRFP‐SRL reporter gene were used for analysis. In these transformants, the globular bodies containing the mRFP‐SRL reporter protein were clearly distinct from normal vacuoles, accumulated the peroxisomal mRFP marker and distorted the GFP‐labelled tonoplast membrane (Fig. 5e,f,i). This phenotype was not confined to the protonemal stage of development. Giant peroxisomes accumulated also in the cells of the gametophore leaves of the mutant compared with the wild type (Fig. 5g–i).

### Phypa_PEX11‐1 accumulates in the peroxisomal membrane

**Figure 6:**
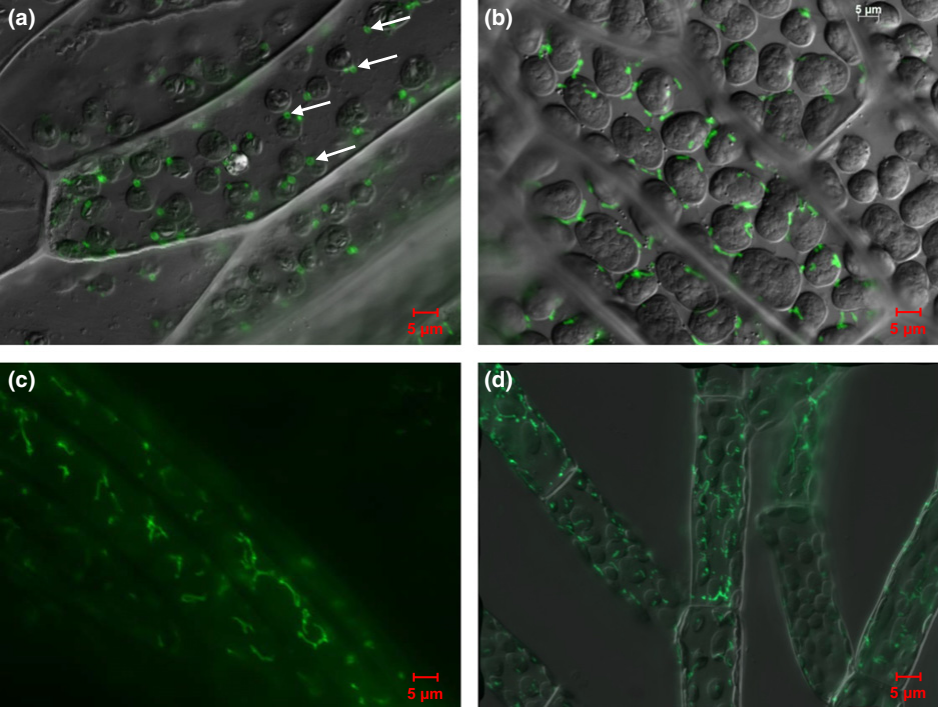
Phypa_PEX11‐1 is located in the peroxisome membrane and overexpression causes tabulation. (a) Merged bright‐field and GFP image of *Physcomitrella patens* gametophore cells showing GFP‐PEX11 localization to the membranes of peroxisomes greatly reduced in size. Peroxisomes showing this particularly clearly are arrowed. (b–d) Tubulation can be seen in GFP (b) and merged (c, d) images of gametophore (c) and chloronemal (d) cells overexpressing the *GFP‐Phypa_PEX11‐1* gene.

PEX11 is a peroxisomal membrane protein that coordinates the assembly and division of the peroxisomes through interactions with members of the Fis1 family to recruit dynamin to mediate the fission of peroxisomal tubules into smaller bodies. Transient expression of GFP‐PEX11‐1 in cells of the *Phypa_pex11‐1‐KO* mutant line complemented the mutant phenotype and reduced the size of the peroxisomes in the transformed cells (Figs 6, S5). Variability inherent in the microprojectile bombardment procedure generates cells expressing the transgene to different extents. The localization of the GFP‐tagged PEX11‐1 to the peroxisomal membrane could be observed through the identification of ‘doughnut’‐like structures (Fig. 6a) in some cells, while in many cells, transient over‐expression of the PEX11 fusion protein resulted in a characteristic PEX11 overexpression phenotype, with extensively tubulated structures accumulating (Fig 6b–d). Co‐transformation of *P. patens* protoplasts with GFP‐PEX11 and mRFP‐SRL confirmed faithful targeting of GFP‐PEX11 to peroxisome membranes (Fig. S5).

**Figure 7:**
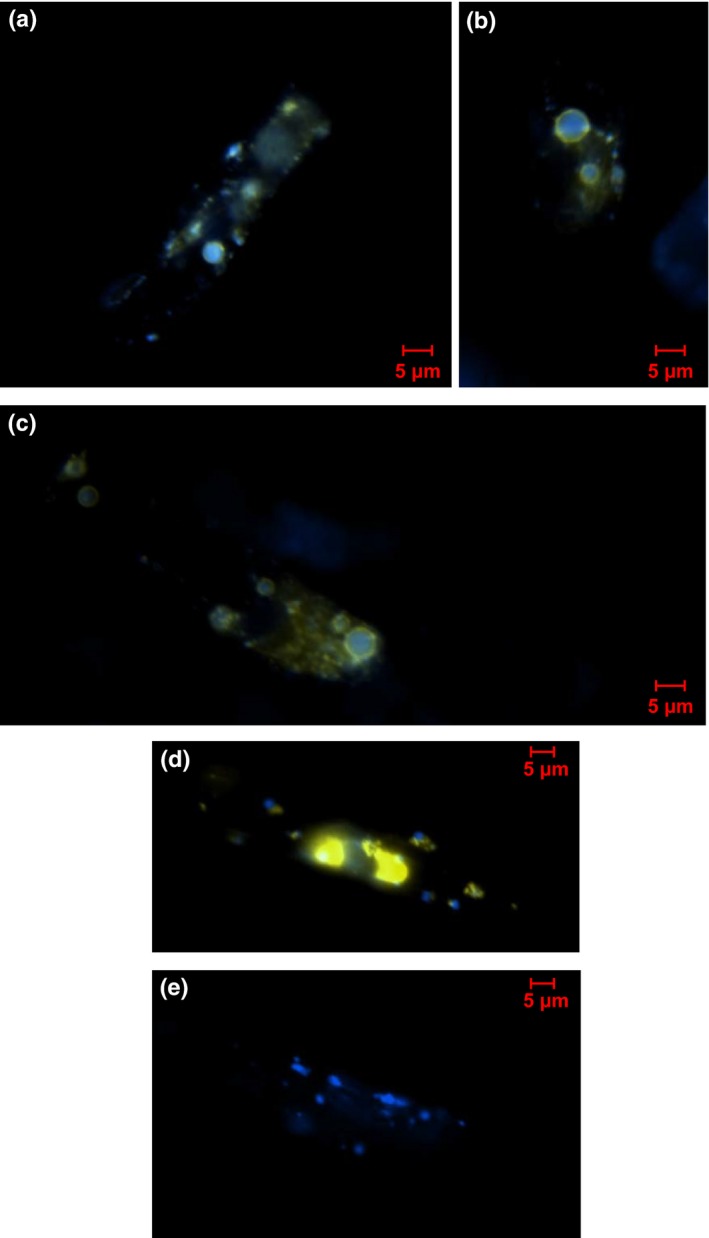
*Physcomitrella patens* PEX11‐1 interacts with fission factors. (a–c) Protonemal tissue of the *Phypa_pex11‐1‐KO* mutant expressing a CFP‐SKL transgene was co‐bombarded with a *Phypa_PEX11‐* *YFP*_*N*_ fusion construct and a *PpFIS1A‐* *YFP*_*C*_ fusion construct. YFP fluorescence detected by epifluorescence microscopy is clearly visible around the periphery of the CFP‐marked peroxisomes, indicating an interaction between the Pex11 and Fission 1A (Fis1A) proteins at the peroxisome membrane. (d) Control experiment in which the CFP‐SKL expressing strain was co‐bombarded with an unfused *YFP*_*N*_ construct and a *PpFIS1A‐* *YFP*_*C*_ construct. (e) Control experiment in which the CFP‐SKL expressing strain was co‐bombarded with an unfused *YFP*_*C*_ construct and a *Phypa_PEX11‐1‐* *YFP*_*N*_ fusion construct. Each panel in this figure shows the merged YFP and CFP fluorescence signal.

We were also able to demonstrate an interaction between Phypa_PEX11‐1 and the fission factors Phypa_Fis1A and Phypa_Fis1B *in vivo*, by BiFC. A stably transformed *Phypa_pex11‐1‐KO* line expressing a CFP‐SKL fusion as a peroxisome marker was transiently transformed by particle co‐bombardment, with constructs expressing the N‐terminal sequence of YFP (YFP_N_) and the C‐terminal sequence of YFP (YFP_C_) fused with *Phypa_PEX11‐1* and *PpFIS1A* and *PpFIS1B* in various combinations. Fig. 7(a–c) illustrates how co‐transformation with a *Phypa_PEX11‐YFP*_*N*_ construct and a *PpFIS1A‐YFP*_*C*_ construct enabled the visualization of YFP fluorescence decorating the perimeters of the CFP‐loaded peroxisomes. By contrast, in control experiments (in which the YFP_N_ sequence was not fused to the Phypa_PEX11‐1 sequence), no YFP co‐localization with peroxisomes was observed, although nonspecific aggregates occasionally occurred (Fig. 7d). When *Phypa_PEX11‐1‐YFP*_*N*_ was expressed with unfused YFPc, no fluorescence was seen (Fig. 7e). Similar results were obtained for interactions between Phypa_PEX11‐1 and Phypa_Fis1B (Figs 6, S6).

### Formation of giant peroxisomes appears growth‐related

**Figure 8:**
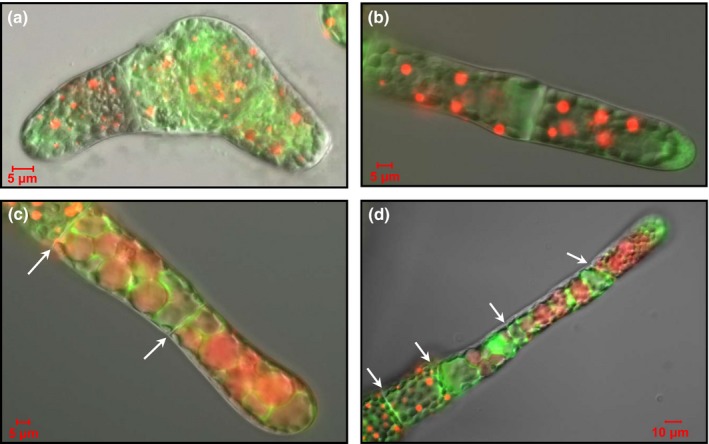
Recovery from low temperature. Protonemata of the *Physcomitrella patens pex11‐1‐KO* line expressing the mRFP‐SRL and AtVam3‐GFP reporters that had been archived at low temperature for over 6 months were homogenized and the homogenate fragments subcultured on cellophane‐overlaid agar medium for regeneration under standard growth conditions. Merged bright‐field, GFP and RFP epifluorescence images of protonemata regenerated for 1 (a), 2 (b) and 4 (c, d) d are shown. The cell walls dividing the filament are indicated by arrows in (c) and (d). The image in (c) contains two complete cells and a part of a third (the antepenultimate cell for the filament), while (d) contains four cells and part of a fifth. The cell wall between the apical and subapical cell in this filament is slightly oblique, indicating that the apical cell is commencing differentiation into a caulonemal initial. Note also the smaller peroxisomes in the older cells of each filament.

During the course of our investigation, we made an interesting observation. Routine maintenance of *P. patens* strains involves incubation at low temperature (7°C) and low light (2 h of illumination every 24 h) for the medium‐ to long‐term storage of cultures. We noticed that, after prolonged storage under these conditions, protonemal homogenate cultures of *Phypa_pex11‐1‐KO* mutant strains no longer contained giant organelles within their cells. However, upon subculture and growth under standard conditions (25°C; continuous illumination), the giant organelle phenotype was rapidly re‐established. This could be clearly seen when protonemal tissue recovered from long‐term low‐temperature storage was homogenized and propagated on cellophane overlays. Using the transgenic strain expressing the AtVAM3 and GFP‐SKL markers, we were able to observe a steady increase in the size of the peroxisomes within protonemata during the first few days of subculture (Fig. 8). The increase in peroxisome size occurred in newly formed cells. Observations of cells along the length of each filament showed that the oldest cells (at the base of each filament, and probably corresponding to cells that had been maintained in storage at low temperature) contained relatively small peroxisomes, whereas the younger cells (the apical and subapical cells in the 4‐d‐old tissue shown in Fig. 8c,d) would have been generated by successive mitosis of the apical cell of each filament following transfer to standard growth conditions, and these contained significantly larger peroxisomes. The filament in Fig. 8(d) contained three new cells, with the apical cell starting to differentiate into a caulonemal cell initial (indicated by the oblique angle of the cell wall separating it from the subapical cell). As the cell cycle progression of chloronemal apical cells is relatively slow (*c*. 24 h), the production of three new cells, containing greatly enlarged peroxisomes, in the first 4 d following return to standard growth conditions suggests a link between growth rate and the demand for peroxisomal biogenesis.

## Discussion

The *PEX11* family in *P. patens*, as in other plant species, is comprised of a number of paralogues, four of which fall into the same clade as Arabidopsis PEX11a and b and two of which fall into the same clade as AtPEX11c, d and e. One gene (*Phypa_PEX11‐1*) is expressed at a level > 4000 times higher than that of the other member of the PEX11cde clade (*Phypa_PEX11‐2*) and also more than an order of magnitude higher than those of the four genes of the PEX11 a/b clade (*Phypa_PEX11‐3‐4‐5‐6*).

The presence of paralogous genes with at least partially redundant function has hindered the analysis of plant PEX11 function through the analysis of knockout mutants. Two groups have produced transgenic Arabidopsis plants where *pex11a* and *b* on the one hand or *pex11c, d* or *e* on the other have been down‐regulated by RNA interference (RNAi). (Nito *et al*., 2007) reported a modest increase in peroxisome size; the peroxisomes in the *pex11a/bi* lines had an average diameter of 1.5 μm while those in the *pex11c/d/e* in lines had an average diameter of 2.36 μm. Orth *et al*. (2007) reported that in their RNAi lines there was a strong reduction of peroxisome number (< 75% compared with control plants), but either no change or a reduction in peroxisome size. The RNAi lines did not show any alteration in growth or peroxisome function. However, it should be noted that in both studies there was still detectable transcript for all isoforms. By contrast, the *Phypa_pex11‐1KO* mutant in which only a single gene was disrupted grew more slowly than wild type as a result of reduced cell elongation (Fig. 3) and the developmental transition from a primary filamentous stage (‘juvenile’) to the development of gametophores (‘adult’) was retarded (Fig. 2). This may be because the mutant line did not produce any PEX11 immunoreactive protein (Fig. 1d) and may be closer to a true knockout of the *PEX11cde* clade than the Arabidopsis RNAi lines.

These mutants produced giant peroxisomes of up to 10 μm in diameter which were readily visible in the light microscope as apparently ‘empty’ regions of the cells (Fig. 4). These structures were confirmed as peroxisomes as they imported the peroxisomal matrix proteins GFP‐SKL and mRFP‐SRL and their membranes were decorated with a *GFP‐Phypa_PEX11‐1* fusion protein (Figs 4, 5, 6, S5). Transient overexpression of *GFP‐Phypa_PEX11‐1* reverted the giant peroxisome phenotype and resulted in the formation of elongated peroxisomes, as has been reported for overexpression of *PEX11* from other organisms.

Comparison of the sequences of the moss PEX11 family with other homologues shows strong conservation of the amphipathic helical structure found in the N terminus of PEX11 in other species (Figs S2–S4), which has been shown to insert into membranes *in vitro* and cause tubulation (Opalinski *et al*., 2011). A recent publication reported that this region of *Hansenula polymorpha* Pex11 interacts directly with Dmn1p (equivalent of Drp in yeasts and DLP in humans) and acts as a GTPase activating protein (GAP) to promote severing of tubulated peroxisomes. HsPex11β also had GAP activity but this was restricted to the first 12 amino acids (helix 1) rather than the amphipathic helix (Williams *et al*., 2015). Comparison of sequence alignments between Phypa_PEX11‐1, HsPEX11β, AtPEX11d and HpPEX11 showed that the region from 55 to 67 within HpPEX11 that is critical for GAP activity is absent in the plant homologues but that the character and spacing of amino acids in the helix 1 motif are conserved, raising the possibility that this region could also have similar activity in plants. Thus, it seems likely that PEX11 functions similarly in peroxisome proliferation and division across kingdoms.

The enlarged peroxisome phenotype seen in the *P. patens* knockout is more reminiscent of that seen in the *S. cerevisiae pex11Δ* mutant (Erdmann & Blobel, 1995) than that in Arabidopsis. *Saccharomyces cerevisiae* has only one *PEX11* gene (although distinct but related genes *PEX25* and *PEX27* also play a role in peroxisome proliferation (Rottensteiner *et al*., 2003; Vizeacoumar *et al*., 2003)) so this similarity may reflect a more complete knock‐out of PEX11 function in the moss compared with the Arabidopsis RNAi lines. Alternatively, it could suggest a greater functional specialization of PEX11 family members in *P. patens*, such that the remaining isoforms cannot rescue the phenotype to the same extent.

A search of the *P. patens* genome sequence also identified homologues of Fis1a and b (Zhang & Hu, 2008) and dynamin‐related proteins (DRPs) 3A (Mano *et al*., 2004) and 3B and 5B (Zhang & Hu, 2010), which are involved in peroxisome division in other organisms. A recent publication has demonstrated the redundant role of members of the DRP5B family in chloroplast division in *P. patens* (Sakaguchi *et al*., 2011) but peroxisome phenotypes were not investigated. To bring about peroxisome division, PEX11 proteins interact with Fis1, which is dual targeted to peroxisomes and mitochondria and recruits the DRPs for organelle division. The interaction between Fis1 and Pex11 is also conserved in *P. patens*, as shown by BiFC (Fig. 7).

Intriguingly, the ‘giant peroxisome’ phenotype is environmentally determined. Upon storage at low temperature with very short day length, the peroxisomes in the *Phypa_pex11‐1 KO* reverted to a more normal size but rapidly increased again in rapidly growing cells upon transfer to longer day length and warmer temperatures (Fig. 8). It is noteworthy that in Arabidopsis light, via *PEX11b*, brings about peroxisome proliferation (Hu & Desai, 2008). It will be interesting to determine whether one of the six *PEX11a/b* homologues is required for light‐dependent increases in peroxisomes in *P. patens*, although evidently none of the other *PEX11* genes can substitute for the role in division.

The conservation of matrix and membrane protein import pathways, the ease with which *P. patens* can be used to generate gene disruptions or allele replacements and the uncoupling of peroxisome growth and division in the *Phypa_pex11‐1* mutant make this a powerful system in which to explore mechanisms of peroxisome biogenesis and the study of peroxisome biology more generally in photosynthetic organisms.

## Author contributions

A.B., A.C.C., C.D.K. and Y.K. planned and designed the research, Y.K., S.M. and M.E‐S. performed experiments, Y.K., S.M., A.C.C. and A.B. analysed data, and A.B., A.C.C. and Y.K. wrote the manuscript. All authors read and approved the final version.

## Supporting information

Please note: Wiley Blackwell are not responsible for the content or functionality of any supporting information supplied by the authors. Any queries (other than missing material) should be directed to the *New Phytologist* Central Office.

**Fig. S1** Plasmids used for gene targeting and reporter fusions.
**Fig. S2** PEX11AB multiple sequence alignment.
**Fig. S3** PEX11CDE multiple sequence alignment.
**Fig. S4** Conservation of sequence features of PEX11 between diverse organisms.
**Fig. S5** PpPEX11‐1 localizes to the peroxisomal membrane.
**Fig. S6** *P. patens pex11‐KO* strain co‐bombarded with pCFP‐SKL+ pYFPn‐Pex11 +  YFPc‐Fis1b.
**Table S1** Gene and protein IDs of *PhypaPEX11* genes
**Table S2** Primers used for PCR amplifications
**Table S3** Digital gene expression analysis of *Phypa_PEX11* family members

## References

[nph13739-bib-0001] Abramoff M , Magalhaes P , Ram S . 2004 Processing with ImageJ. Biophotonics International 11: 36–42.

[nph13739-bib-0002] Barton K , Mathur N , Mathur J . 2013 Simultaneous live‐imaging of peroxisomes and the ER in plant cells suggests contiguity but no luminal continuity between the two organelles. Frontiers in Physiology 4: 196. 23898304 10.3389/fphys.2013.00196 PMC3721060

[nph13739-bib-0003] Bonekamp NA , Grille S , Cardoso MJ , Almeida M , Aroso M , Gomes S , Magalhaes AC , Ribeiro D , Islinger M , Schrader M . 2013 Self‐interaction of human Pex11p beta during peroxisomal growth and division. PLoS ONE 8: e53424. 23308220 10.1371/journal.pone.0053424 PMC3538539

[nph13739-bib-0004] Chang J , Klute MJ , Tower RJ , Mast FD , Dacks JB , Rachubinski RA . 2015 An ancestral role in peroxisome assembly is retained by the divisional peroxin Pex11 in the yeast *Yarrowia lipolytica* . Journal of Cell Science 128: 1327–1340. 25663700 10.1242/jcs.157743

[nph13739-bib-0005] Desai M , Hu J . 2008 Light induces peroxisome proliferation in Arabidopsis seedlings through the photoreceptor phytochrome A, the transcription factor HY5 HOMOLOG, and the peroxisomal protein PEROXIN11b. Plant Physiology 146: 1117–1127. 18203870 10.1104/pp.107.113555 PMC2259046

[nph13739-bib-0006] Ebberink MS , Koster J , Visser G , van Spronsen F , Stolte‐Dijkstra I , Smit GPA , Fock JM , Kemp S , Wanders RJA , Waterham HR . 2012 A novel defect of peroxisome division due to a homozygous non‐sense mutation in the PEX11 beta gene. Journal of Medical Genetics 49: 307–313. 22581968 10.1136/jmedgenet-2012-100778

[nph13739-bib-0007] Erdmann R , Blobel G . 1995 Giant peroxisomes in oleic acid‐induced *Saccharomyces cerevisiae* lacking the peroxisomal membrane‐protein Pmp27p. Journal of Cell Biology 128: 509–523. 7860627 10.1083/jcb.128.4.509 PMC2199900

[nph13739-bib-0008] Fagarasanu A , Fagarasanu M , Rachubinski RA . 2007 Maintaining peroxisome populations: a story of division and inheritance. Annual Review of Cell and Developmental Biology 23: 321–344. 10.1146/annurev.cellbio.23.090506.123456 17506702

[nph13739-bib-0009] Ferreira RMB , Bird B , Davies DD . 1989 The effect of light on the structure and organization of *Lemna* peroxisomes. Journal of Experimental Botany 40: 1029–1035.

[nph13739-bib-0010] Furt F , Lemoi K , Tuzel E , Vidali L . 2012 Quantitative analysis of organelle distribution and dynamics in *Physcomitrella patens* protonemal cells. BMC Plant Biology 12: 70. 22594499 10.1186/1471-2229-12-70 PMC3476433

[nph13739-bib-0011] Gurvitz A , Hiltunen JK , Erdmann R , Hamilton B , Hartig A , Ruis H , Rottensteiner H . 2001 *Saccharomyces cerevisiae* Adr1p governs fatty acid beta‐ oxidation and peroxisome proliferation by regulating *POX1* and *PEX11* . Journal of Biological Chemistry 276: 31825–31830. 11431484 10.1074/jbc.M105989200

[nph13739-bib-0012] Gurvitz A , Rottensteiner H . 2006 The biochemistry of oleate induction: transcriptional upregulation and peroxisome proliferation. Biochimica et Biophysica Acta (BBA) – Molecular Cell Research 1763: 1392. 16949166 10.1016/j.bbamcr.2006.07.011

[nph13739-bib-0013] Hettema EH , Erdmann R , van der Klei I , Veenhuis M . 2014 Evolving models for peroxisome biogenesis. Current Opinion in Cell Biology 29: 25–30. 24681485 10.1016/j.ceb.2014.02.002 PMC4148619

[nph13739-bib-0014] Hu J , Desai M . 2008 Light control of peroxisome proliferation during Arabidopsis photomorphogenesis. Plant Signaling & Behavior 3: 801–803. 19704562 10.4161/psb.3.10.5876 PMC2634377

[nph13739-bib-0015] Huang CY , Chung CI , Lin YC , Hsing YIC , Huang AHC . 2009 Oil bodies and oleosins in *Physcomitrella* possess characteristics representative of early trends in evolution. Plant Physiology 150: 1192–1203. 19420327 10.1104/pp.109.138123 PMC2705038

[nph13739-bib-0016] Joshi S , Agrawal G , Subramani S . 2012 Phosphorylation‐dependent Pex11p and Fis1p interaction regulates peroxisome division. Molecular Biology of the Cell 23: 1307–1315. 22337771 10.1091/mbc.E11-09-0782 PMC3315806

[nph13739-bib-0017] Kamisugi Y , Cuming AC , Cove DJ . 2005 Parameters determining the efficiency of homologous recombination mediated gene targeting in the moss *Physcomitrella patens* . Nucleic Acids Research 33: e173. 16282584 10.1093/nar/gni172 PMC1283530

[nph13739-bib-0018] Kamisugi Y , Schaefer DG , Kozak J , Charlot F , Vrielynck N , Hola M , Angelis KJ , Cuming AC , Nogue F . 2012 MRE11 and RAD50, but not NBS1, are essential for gene targeting in the moss *Physcomitrella patens* . Nucleic Acids Research 40: 3496–3510. 22210882 10.1093/nar/gkr1272 PMC3333855

[nph13739-bib-0019] Kamisugi Y , Schlink K , Rensing SA , Schween G , von Stackelberg M , Cuming AC , Reski R , Cove DJ . 2006 The mechanism of gene targeting in *Physcomitrella patens*: homologous recombination, concatenation and multiple integration. Nucleic Acids Research 34: 6205–6214. 17090599 10.1093/nar/gkl832 PMC1693892

[nph13739-bib-0020] Kaur N , Hu JP . 2009 Dynamics of peroxisome abundance: a tale of division and proliferation. Current Opinion in Plant Biology 12: 781–788. 19734083 10.1016/j.pbi.2009.08.001

[nph13739-bib-0021] van der Klei IJ , Yurimoto H , Sakai Y , Veenhuis M . 2006 The significance of peroxisomes in methanol metabolism in methylotrophic yeast. Biochimica et Biophysica Acta (BBA) – Molecular Cell Research 1763: 1453. 17023065 10.1016/j.bbamcr.2006.07.016

[nph13739-bib-0022] Knight CD , Cove DJ , Cuming AC , Quatrano RS . 2002 Moss gene technology In: Gilmartin P, Bowler C, eds. Molecular plant biology. Oxford, UK: Oxford University Press, 285–299.

[nph13739-bib-0023] Knoblach B , Rachubinski RA . 2010 Phosphorylation‐dependent activation of peroxisome proliferator protein PEX11 controls peroxisome abundance. Journal of Biological Chemistry 285: 6670–6680. 20028986 10.1074/jbc.M109.094805 PMC2825462

[nph13739-bib-0024] Kobayashi S , Tanaka A , Fujiki Y . 2007 Fis1, DLP1, and Pex11p coordinately regulate peroxisome morphogenesis. Experimental Cell Research 313: 1675–1686. 17408615 10.1016/j.yexcr.2007.02.028

[nph13739-bib-0025] Koch J , Pranjic K , Huber A , Ellinger A , Hartig A , Kragler F , Brocard C . 2010 PEX11 family members are membrane elongation factors that coordinate peroxisome proliferation and maintenance. Journal of Cell Science 123: 3389–3400. 20826455 10.1242/jcs.064907

[nph13739-bib-0026] Li XL , Baumgart E , Dong GX , Morrell JC , Jimenez‐Sanchez G , Valle D , Smith KD , Gould SJ . 2002a PEX11 alpha is required for peroxisome proliferation in response to 4‐phenylbutyrate but is dispensable for peroxisome proliferator‐activated receptor alpha‐mediated peroxisome proliferation. Molecular and Cellular Biology 22: 8226–8240. 12417726 10.1128/MCB.22.23.8226-8240.2002 PMC134051

[nph13739-bib-0027] Li XL , Baumgart E , Morrell JC , Jimenez‐Sanchez G , Valle D , Gould SJ . 2002b PEX11 beta deficiency is lethal and impairs neuronal migration but does not abrogate peroxisome function. Molecular and Cellular Biology 22: 4358–4365. 12024045 10.1128/MCB.22.12.4358-4365.2002 PMC133847

[nph13739-bib-0028] Lingard MJ , Gidda SK , Bingham S , Rothstein SJ , Mullen RT , Trelease RN . 2008 Arabidopsis PEROXIN11c‐e, FISSION1b, and DYNAMIN‐RELATED PROTEIN3A cooperate in cell cycle‐associated replication of peroxisomes. Plant Cell 20: 1567–1585. 18539750 10.1105/tpc.107.057679 PMC2483373

[nph13739-bib-0029] Lingard MJ , Trelease RN . 2006 Five Arabidopsis peroxin 11 homologs individually promote peroxisome elongation, duplication or aggregation. Journal of Cell Science 119: 1961–1972. 16636080 10.1242/jcs.02904

[nph13739-bib-0030] Lopez‐Huertas E , Charlton WL , Johnson B , Graham IA , Baker A . 2000 Stress induces peroxisome biogenesis genes. Embo Journal 19: 6770–6777. 11118212 10.1093/emboj/19.24.6770 PMC305880

[nph13739-bib-0031] Mano S , Nakamori C , Kondo M , Hayashi M , Nishimura M . 2004 An Arabidopsis dynamin‐related protein, DRP3A, controls both peroxisomal and mitochondrial division. Plant Journal 38: 487–498. 15086806 10.1111/j.1365-313X.2004.02063.x

[nph13739-bib-0032] Marshall PA , Dyer JM , Quick ME , Goodman JM . 1996 Redox‐sensitive homodimerization of Pex11p: a proposed mechanism to regulate peroxisomal division. Journal of Cell Biology 135: 123–137. 8858168 10.1083/jcb.135.1.123 PMC2121026

[nph13739-bib-0033] Marshall PA , Krimkevich YI , Lark RH , Dyer JM , Veenhuis M , Goodman JM . 1995 Pmp27 promotes peroxisomal proliferation. Journal of Cell Biology 129: 345–355. 7721939 10.1083/jcb.129.2.345 PMC2199913

[nph13739-bib-0034] Mattiazzi Usaj M , Brloznik M , Kaferle P , Zitnik M , Wolinski H , Leitner F , Kohlwein SD , Zupan B , Petrovic U . 2015 Genome‐wide localization study of Yeast Pex11 identifies peroxisome‐mitochondria interactions through the ERMES complex. Journal of Molecular Biology 427: 2072–2087. 25769804 10.1016/j.jmb.2015.03.004 PMC4429955

[nph13739-bib-0035] Mitsuya S , El‐Shami M , Sparkes IA , Charlton WL , Lousa CD , Johnson B , Baker A . 2010 Salt stress causes peroxisome proliferation, but inducing peroxisome proliferation does not improve NaCl tolerance in *Arabidopsis thaliana* . PLoS ONE 5: e9408. 20195524 10.1371/journal.pone.0009408 PMC2827565

[nph13739-bib-0036] Nayidu NK , Wang L , Xie WB , Zhang CJ , Fan CZ , Lian XM , Zhang QF , Xiong LZ . 2008 Comprehensive sequence and expression profile analysis of *PEX11* gene family in rice. Gene 412: 59–70. 18291602 10.1016/j.gene.2008.01.006

[nph13739-bib-0037] Nito K , Kamigaki A , Kondo M , Hayashi M , Nishimura M . 2007 Functional classification of Arabidopsis peroxisome biogenesis factors proposed from analyses of knockdown mutants. Plant and Cell Physiology 48: 763–774. 17478547 10.1093/pcp/pcm053

[nph13739-bib-0038] Oda Y , Hirata A , Sano T , Fujita T , Hiwatashi Y , Sato Y , Kadota A , Hasebe M , Hasezawa S . 2009 Microtubules regulate dynamic organization of vacuoles in *Physcomitrella patens* . Plant and Cell Physiology 50: 855–868. 19251746 10.1093/pcp/pcp031

[nph13739-bib-0039] Opalinski L , Kiel J , Williams C , Veenhuis M , van der Klei IJ . 2011 Membrane curvature during peroxisome fission requires Pex11. EMBO Journal 30: 5–16. 21113128 10.1038/emboj.2010.299 PMC3020119

[nph13739-bib-0040] Orth T , Reumann S , Zhang XC , Fan JL , Wenzel D , Quan S , Hu JP . 2007 The PEROXIN11 protein family controls peroxisome proliferation in Arabidopsis. Plant Cell 19: 333–350. 17220199 10.1105/tpc.106.045831 PMC1820951

[nph13739-bib-0041] Palma JM , Gomez M , Yanez J , Del Rio LA . 1987 Increased levels of peroxisomal active oxygen‐related enzymes in copper‐tolerant pea plants. Plant Physiology 85: 570–574. 16665737 10.1104/pp.85.2.570 PMC1054296

[nph13739-bib-0042] Pracharoenwattana I , Cornah JE , Smith SM . 2005 Arabidopsis peroxisomal citrate synthase is required for fatty acid respiration and seed germination. Plant Cell 17: 2037–2048. 15923350 10.1105/tpc.105.031856 PMC1167550

[nph13739-bib-0043] Rahim G , Bischof S , Kessler F , Agne B . 2009 *In vivo* interaction between atToc33 and atToc159 GTP‐binding domains demonstrated in a plant split‐ubiquitin system. Journal of Experimental Botany 60: 257–267. 19010773 10.1093/jxb/ern283

[nph13739-bib-0044] Rensing SA , Lang D , Zimmer AD , Terry A , Salamov A , Shapiro H , Nishiyama T , Perroud PF , Lindquist EA , Kamisugi Y *et al* 2008 The *Physcomitrella* genome reveals evolutionary insights into the conquest of land by plants. Science 319: 64–69. 18079367 10.1126/science.1150646

[nph13739-bib-0045] van Roermund CWT , Tabak HF , van den Berg M , Wanders RJA , Hettema EH . 2000 Pex11p plays a primary role in medium‐chain fatty acid oxidation, a process that affects peroxisome number and size in *Saccharomyces cerevisiae* . Journal of Cell Biology 150: 489–497. 10931862 10.1083/jcb.150.3.489 PMC2175187

[nph13739-bib-0046] Rottensteiner H , Stein K , Sonnenhol E , Erdmann R . 2003 Conserved function of Pex11p and the novel Pex25p and Pex27p in peroxisome biogenesis. Molecular Biology of the Cell 14: 4316–4328. 14517338 10.1091/mbc.E03-03-0153 PMC207022

[nph13739-bib-0047] Sakaguchi E , Takechi K , Sato H , Yamada T , Takio S , Takano H . 2011 Three dynamin‐related protein 5B genes are related to plastid division in *Physcomitrella patens* . Plant Science 180: 789–795. 21497715 10.1016/j.plantsci.2011.02.003

[nph13739-bib-0048] Sanford JC , Smith FD , Russell JA . 1993 Optimizing the biolistic process for different biological applications. Methods in Enzymology 217: 483–509. 8474348 10.1016/0076-6879(93)17086-k

[nph13739-bib-0049] Schaefer D , Zryd J . 1997 Efficient gene targeting in the moss *Physcomitrella patens* . Plant Journal 11: 1195–1206. 9225463 10.1046/j.1365-313x.1997.11061195.x

[nph13739-bib-0050] Schrader M . 2006 Shared components of mitochondrial and peroxisomal division. Biochimica Et Biophysica Acta‐Molecular Cell Research 1763: 531–541. 10.1016/j.bbamcr.2006.01.004 16487606

[nph13739-bib-0051] Schrader M , Bonekamp NA , Islinger M . 2012 Fission and proliferation of peroxisomes. Biochimica et Biophysica Acta–Molecular Basis of Disease 1822: 1343–1357. 10.1016/j.bbadis.2011.12.014 22240198

[nph13739-bib-0052] Schrader M , Reuber BE , Morrell JC , Jimenez‐Sanchez G , Obie C , Stroh TA , Valle D , Schroer TA , Gould SJ . 1998 Expression of PEX11 beta mediates peroxisome proliferation in the absence of extracellular stimuli. Journal of Biological Chemistry 273: 29607–29614. 9792670 10.1074/jbc.273.45.29607

[nph13739-bib-0053] Sparkes IA , Hawes C , Baker A . 2005 AtPEX2 and AtPEX10 are targeted to peroxisomes independently of known endoplasmic reticulum trafficking routes. Plant Physiology 139: 690–700. 16169966 10.1104/pp.105.065094 PMC1255988

[nph13739-bib-0054] Thoms S , Erdmann R . 2005 Dynamin‐related proteins and Pex11 proteins in peroxisome division and proliferation. FEBS Journal 272: 5169–5181. 16218949 10.1111/j.1742-4658.2005.04939.x

[nph13739-bib-0055] Vizeacoumar FJ , Torres‐Guzman JC , Tam YYC , Aitchison JD , Rachubinski RA . 2003 *YHR150w* and *YDR479c* encode peroxisomal integral membrane proteins involved in the regulation of peroxisome number, size, and distribution in *Saccharomyces cerevisiae* . Journal of Cell Biology 161: 321–332. 12707309 10.1083/jcb.200210130 PMC2172915

[nph13739-bib-0056] Whitaker J , Kamisugi Y , Cuming A 2010 The DNA damage response transcriptome of the moss Physcomitrella patens. GSE25237 GA. [WWW document] URL http://www.ncbi.nlm.nih.gov/geo. [accessed 2 October 2015]

[nph13739-bib-0057] Williams C , Opalinski L , Landgraf C , Costello J , Schrader M , Krikken AM , Knoops K , Kram AM , Volkmer R , van der Klei IJ . 2015 The membrane remodeling protein Pex11p activates the GTPase Dnm1p during peroxisomal fission. Proceedings of the National Academy of Sciences, USA 112: 6377–6382. 10.1073/pnas.1418736112 PMC4443378 25941407

[nph13739-bib-0058] Zhang XC , Hu JP . 2008 FISSION1A and FISSION1B proteins mediate the fission of peroxisomes and mitochondria in Arabidopsis. Molecular Plant 1: 1036–1047. 19825601 10.1093/mp/ssn056

[nph13739-bib-0059] Zhang XC , Hu JP . 2009 Two small protein families, DYNAMIN‐RELATED PROTEIN3 and FISSION1, are required for peroxisome fission in Arabidopsis. Plant Journal 57: 146–159. 18785999 10.1111/j.1365-313X.2008.03677.x

[nph13739-bib-0060] Zhang XC , Hu JP . 2010 The Arabidopsis chloroplast division protein DYNAMIN‐RELATED PROTEIN5B also mediates peroxisome division. Plant Cell 22: 431–442. 20179140 10.1105/tpc.109.071324 PMC2845408

