# Supplementary material for: Giant peroxisomes in a moss (Physcomitrella patens) peroxisomal biogenesis factor 11 mutant
Source: New Phytol. 2015 Nov 6;209(2):576–89. doi: 10.1111/nph.13739 (PMC4738463; doi:10.1111/nph.13739)
Supplement: Supplementary file 1 — Fig. S1 Plasmids used for gene targeting and reporter fusions. Fig. S2 PEX11AB multiple sequence alignment. Fig. S3 PEX11CDE multiple sequence alignment. Fig. S4 Conservation of sequence features of PEX11 between diverse organisms. Fig. S5 PpPEX11‐1 localizes to the peroxisomal membrane. Fig. S6 P. patens pex11‐KO strain co‐bombarded with pCFP‐SKL+ pYFPn‐Pex11 + YFPc‐Fis1b. Table S1 Gene and protein IDs of PhypaPEX11 genes Table S2 Primers used for PCR amplifications Table S3 Digital gene expression analysis of Phypa_PEX11 family members [file NPH-209-576-s001.pdf]

New Phytologist Supporting Information

Article title: **Giant peroxisomes in a moss (*Physcomitrella patens*) *pex11* mutant**
Authors: Yasuko Kamisugi, Shiro Mitsuya, Mahmoud El-Shami, Celia D. Knight, Andrew C. Cuming and Alison Baker
Article acceptance date: 01 October 2015

The following Supporting Information is available for this article:

Fig. S1 Plasmids used for gene targeting and reporter fusions.

Fig. S2 PEX11AB multiple sequence alignment.

Fig. S3 PEX11CDE multiple sequence alignment.

Fig. S4 Conservation of sequence features of PEX11 between diverse organisms.

Fig. S5 PpPEX11-1 localises to the peroxisomal membrane.

Fig. S6 *P. patens pex11-KO* strain co-bombarded with pCFP-SKL+ pYFPn-Pex11 + YFPc-Fis1b.

Table S1 Gene and protein IDs of *PhypaPEX11* genes

Table S2 Primers used for PCR amplifications

Table S3 Digital gene expression analysis of *Phypa_PEX11* family members

Fig. S1 Plasmids used for gene targeting and reporter fusions

Figure S1: Plasmids used for gene targeting and reporter fusions

1a: pJHB1: Contains the *pPEX11-1* genomic sequence disrupted by insertion of an *nptII* selection cassette

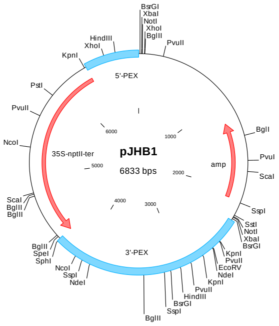

1b :pAmRFP-SRL-H108: Contains the peroxisome-targeted mRFP gene driven by the rice actin-1 promoter. The λH108 sequence targets the construct to a neutral locus

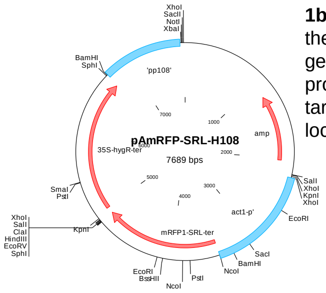

1c: pS65T-cPex11GW: A Pex-11 GFP fusion driven by a 2x 35S promoter and targeted to the λH108 locus

1d: pYFPn-Pex11: Pex-11 cDNA N-terminally fused with the N-terminal half of YFP. For transient expression in BiFC experiments

1e: pYFPc-Fis1a: Fis1a cDNA N-terminally fused with the C-terminal half of YFP. For transient expression in BiFC experiments

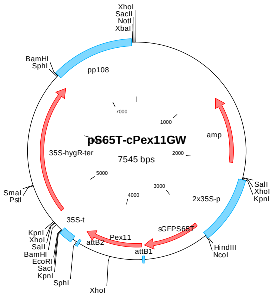

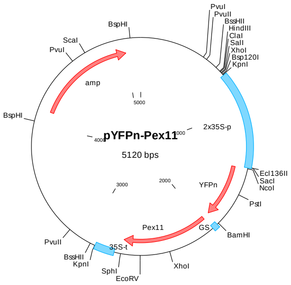

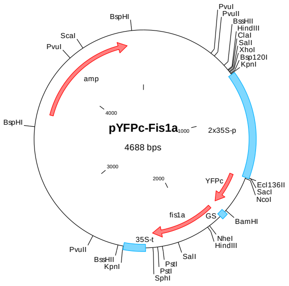

New Phytologist

Fig. S2 PEX11AB multiple sequence alignment

Figure S2: PEX11AB multiple sequence alignment

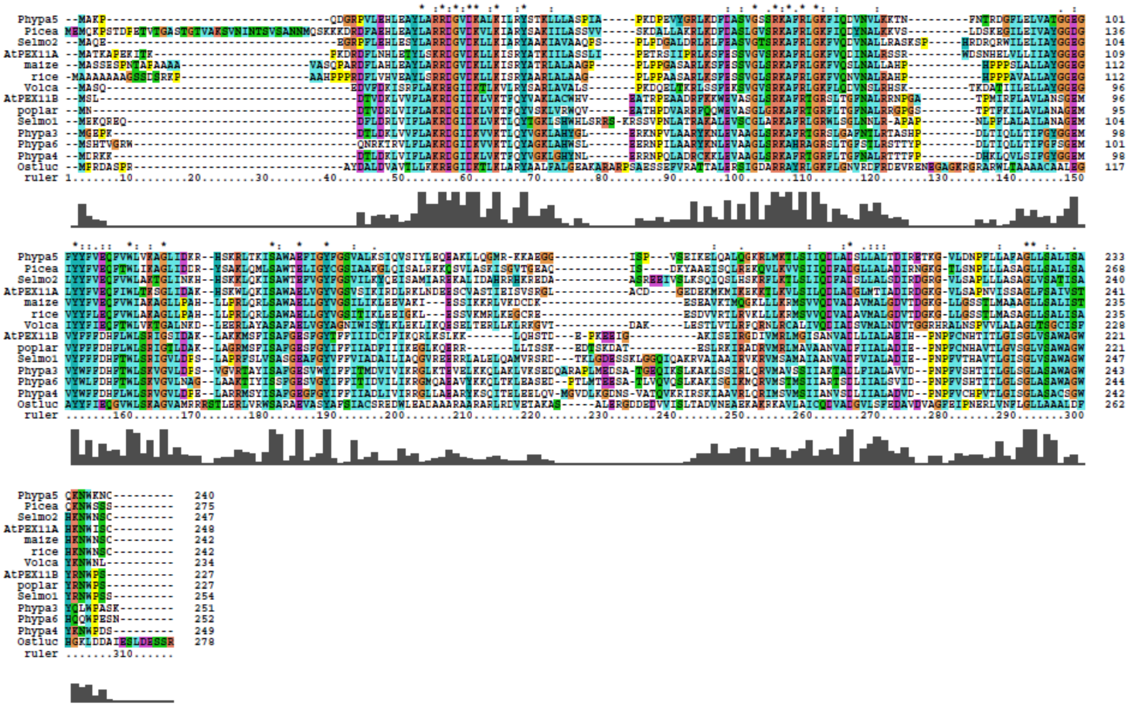

New Phytologist

Fig. S3 PEX11CDE multiple sequence alignment

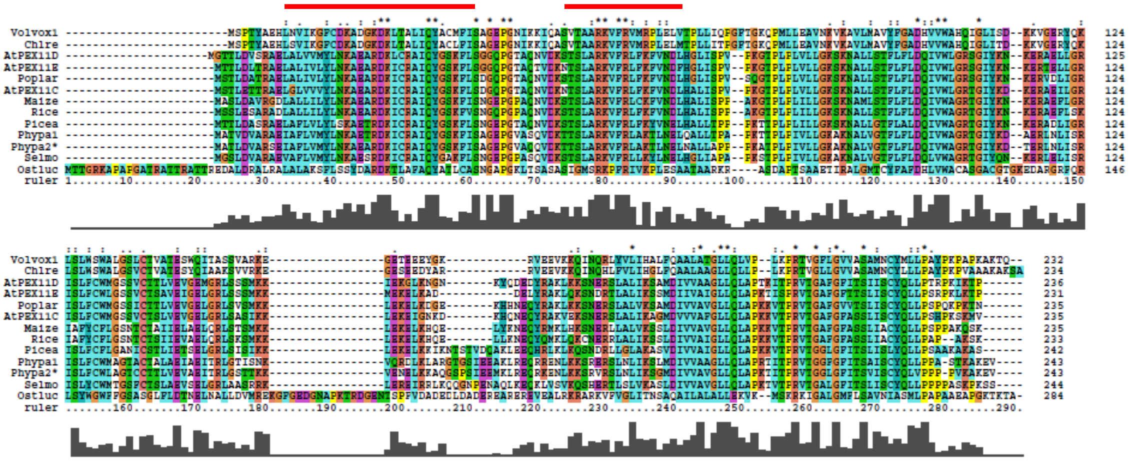

Figure S3: Pex11CDE multiple sequence alignment. The conserved amphipathic helix is overlined in red.

New Phytologist

Fig. S4 Conservation of sequence features of PEX11 between diverse organisms. The conserved amphipathic helical sequences from *Pp*PEX11-1 aligned with the consensus sequence.

Figure S4: Conservation of sequence features of PEX11 between diverse organisms. The conserved amphipathic helical sequences from *Pp*PEX11-1 aligned with the consensus sequence.

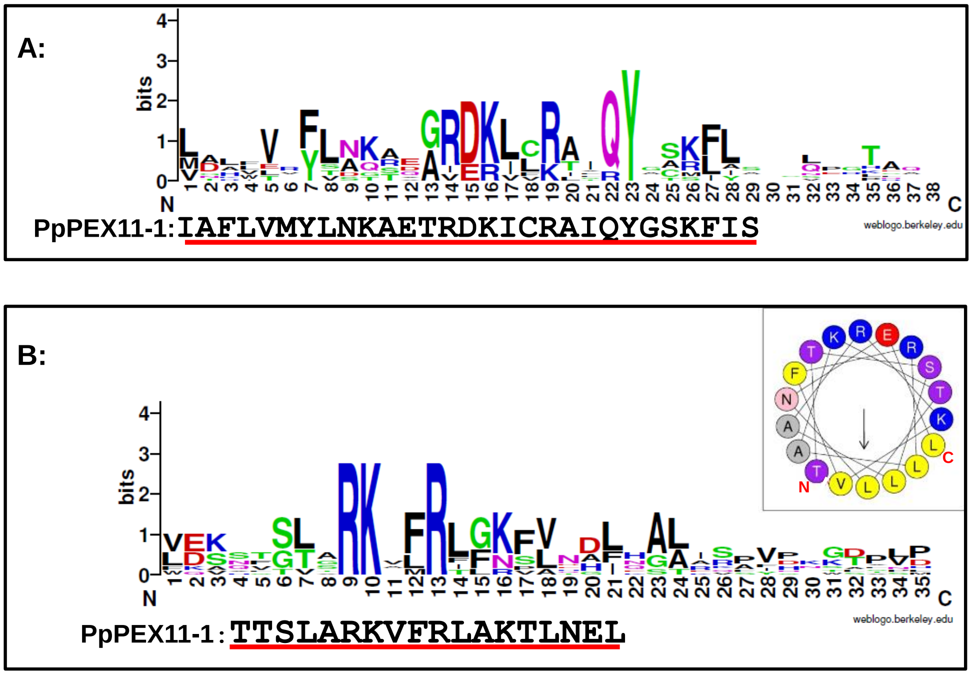

Fig. S5 PpPEX11-1 localises to the peroxisomal membrane. *P. patens* protoplasts were transiently co-transformed with plasmids expressing mRFP-SRL and GFP-PEX11, and fluorescence detected by epifluorescence microscopy (a) Bright-field image; (b) RFP fluorescence; (c) GFP fluorescence; (d) merged image.

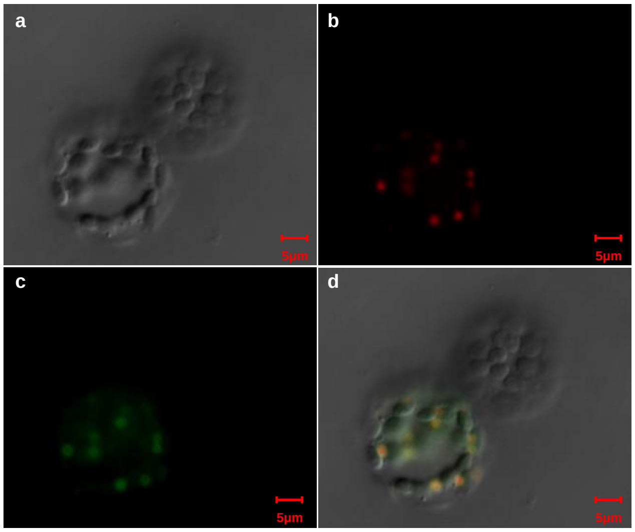

Figure S5: PpPEX11-1 localises to the peroxisomal membrane: *P. patens* protoplasts were transiently co-transformed with plasmids expressing mRFP-SRL and GFP-PEX11, and fluorescence detected by epifluorescence microscopy**a:** Bright-field image; **b:** RFP fluorescence; **c:** GFP fluorescence; **d:** merged image

Fig. S6 *P. patens pex11-KO* strain co-bombarded with pCFP-SKL+ pYFPn-Pex11 + YFPc-Fis1b and viewed by epifluorescence microscopy. (a) CFP fluorescence; (b) YFP fluorescence; (c) CFP and YFP merged image; (d) merged bright-field and fluorescent images. Peroxisomes with interacting Pex11 and Fis1b are arrowed.

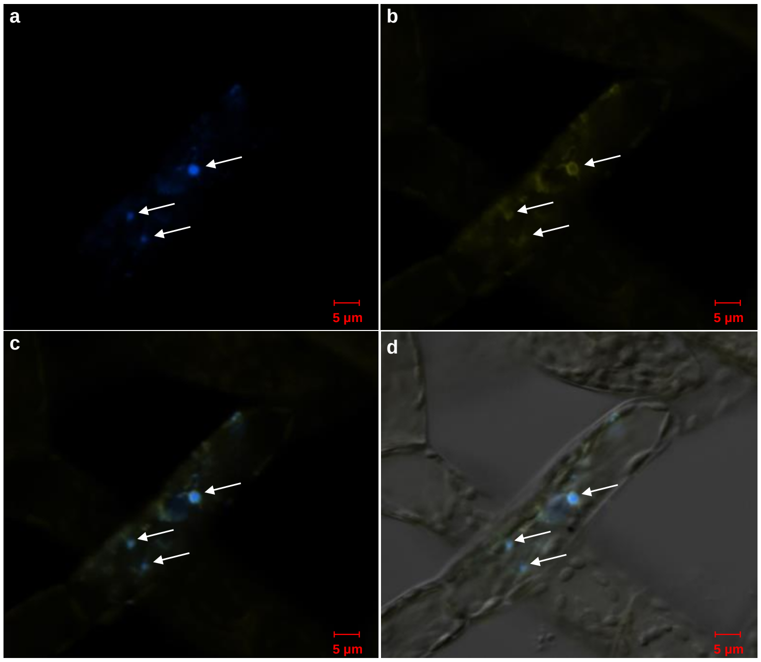

Figure S6: *P. patens pex11-KO* strain co-bombarded with pCFP-SKL+ pYFPn-Pex11 + YFPc-Fis1b and viewed by epifluorescence microscopy. **a:** CFP fluorescence; **b:** YFP fluorescence; **c:** CFP and YFP merged image; **d:** merged bright-field and fluorescent images. Peroxisomes with interacting Pex11 and Fis1b are arrowed.

Table S1 Gene and protein IDs of PhypaPEX11 genes with successive releases of improved assemblies and annotations of the Physcomitrella genome have come revisions of gene and protein IDs. A consequence of this is that with each revision, a new set of IDs have been propagated even when no change in the gene, transcript of protein sequence has been made. This table summarises the alternative identifiers assigned to the PpPEX11 genes in the version 1.1 genome release and annotation (http://genome.jgi-psf.org/pages/search-for-genes.jsf?organism=Phypa1_1), the Version 1.6 genome annotation (https://www.cosmoss.org/fgb2/gbrowse/physcome/), the Version 3.0 genome prerelease (http://phytozome.jgi.doe.gov/pz/portal.html#!search?show=KEYWORD&method=Org_Ppatens) and the V3.1 genome reannotation prerelease (https://www.cosmoss.org/fgb2/gbrowse/V3.1/). Note that for the PpPEX11-2 gene, as noted in the text, only the V3.1 annotation is correct, while for PpPEX11-6 the V3.0 and V3.1 annotations are correct.

***PEX11* gene**	**V1.1**	**V1.6**	**V3.0**	**V3.1**
PpPEX11-1	Phypa1_1:205010	Pp1s433_22V6 (Phypa_460481)	Phpat.019G070900.1	Pp3c19_20730V1.1
PpPEX11-2	Phypa1_1:55082	Pp1s3_624V6.1 (Phypa_422942)	Phpat.018G038300	Pp3c18_11990V1.1
PpPEX11-3	Phypa1_1:63102	Pp1s159_21V6 (Phypa_447344)	Phpat.026G017000.1	Pp3c26_4230V1.1
PpPEX11-4	Phypa1_1:62335	Pp1s16_338V6 (Phypa_426510)	Phpat.024G044700.1	Pp3c24_12360V1.1
PpPEX11-5	Phypa1_1:80254	Pp1s84_298V6 (Phypa_439417)	Phpat.002G050500.2	Pp3c2_11370V1.1
PpPEX11-6	Phypa1_1:118714	Pp1s25_244V6 (Phypa_428462)	Phpat.003G059500	Pp3c3_15780V1.1

Table S2 Primers used for PCR amplifications

Use	Primer	Sequence
rtPCR of Phypa_Pex11-2	PpPex11_Sc3S2	AAGTGGAGCGTGTGGTTCATGT
	PpPex11_Sc3A3	CCCACGACAAATTGCATGTAGA
rtPCR of Phypa_Pex11-6	PpPex11_Sc25F2	TCAAGCAAGCATGTGAATTGGT
	PpPex11_Sc25A2	TCAGAAGGAAATACGTCGAGAGC
Gateway cloning of *Phypa_Pex11* fragment for disruption, and analysis of targeted locus.	p22 (attB2 tail)	GGGGACCACTTTGTACAAGAAAGCTGGGTAAATGCTCACTGATGTTATC
	p21 (attB1 tail)	GGGGACAAGTTTGTACAAAAAAGCAGGCTAGGGTTCACTGAGATTGG
	p4 (PpPex11S)	ATGCTCATACAAATGCAGGTCCAC
	p5 (35Spro2R)	AGATAGCTGGGCAATGGAATCCGA
	p6 (g6termF)	ATTGGTATCAGAGCCATGAATAGGT
Amplification of disrupted fragment for transformation	p2 (PpPex11KOR)	GCGTTGTAGGGAATCTTTTTGGTC
	p3 (PpPex11KOF)	ACGTGACCGTTAGCTCGTAGATCA
Construction of pAct-p	pActinS	CTCTGCAGTCGGTACCTGTTTAGTTCTCGAGGTCA
	pActinNcoAS	CGTCAGCCATGGTCTACCTACAAAAAAGCTCCGCA
Amplification of Physcomitrella genomic locus pp108	pp108F_Bam	TGACAATCGGATCCATGAAAAGACTCGTGCACATGGT
	pp108R_Bgl	TCCTCTGTAGATCTGTCTCGGTAGATCCCTCTTGGAA
	pp108F2_Bam	TTACGTTTGGATCCCTCCACCATGAGCAAC
Construction of GFP-*Phypa_Pex11* (GateWay)	PpPex11_GS	ACAAGTTTGTACAAAAAAGCAGGCTATGGCGACAGTCGATGTTGC
	PpPex11_GA	ACCACTTTGTACAAGAAAGCTGGGTGACCTCCTTAGCCTTGGTTGA
GS linker	G3S1_linker_S	CTCGGTGGAGGCGGTTCAGGCGGAGGTGGCTCTGGCGGTGGCGGATCGGAT
	G3S1_linker_A	ATCCGATCCGCCACCGCCAGAGCCACCTCCGCCTGAACCGCCTCCACCGAG
Construction of BiFC plasmids	Cerl-1SNco	CTATATCGATCCATGGTGAGCAAGGGCGAGGA
	YFPn_ASph	TTTCAGCGGCATGCTCAGATATCGGATCCGGCGGTGATATAGACGTTGTGG
	YFPc_SNco	CTATATCGATCCATGGACAAGCAGAAGAACGGCATC
	S65T-717ABam	GCAGGTCGGATCCCTTGTACAGCTCGTCCATGCC
	cPpPex11_S	ATGGCGACAGTCGATGTTGC
	cPpPex11_A	GACCTCCTTAGCCTTGGTTGA
	cPpFis1a_S	ATGGCAGAAGGATTTTTCAATAAAG
	cPpFis1a_A	TCTCTTGAGACCTCCGAGAACTG
	cPpFis1b_S	ATGGCGGAAGCATTTTTCAACAACA
	cPpFis1b_A	TCTCCTGCGACTGCCAATTACA

New Phytologist

Table S3 Digital gene expression analysis of *Phypa_PEX11* family members. Complementary DNA derived from polyribosomal RNA from 7-d-old chloronemal tissue was subjected to Illumina short-read massively parallel sequencing. The sequence reads were aligned with the *Physcomitrella* genome assembly and the numbers of reads corresponding to each gene model counted. (Whitaker *et al.*, 2010). **Whitaker J, Kamisugi Y, Cuming A. 2010**. The DNA damage response transcriptome of the moss *Physcomitrella patens*. In GSE25237 GA. http://www.ncbi.nlm.nih.gov/geo.

Gene	No. sequence reads
Phypa_PEX11-1	11651
Phypa_PEX11-2	3
Phypa_PEX11-3	1006
Phypa_PEX11-4	780
Phypa_PEX11-5	668
Phypa_PEX11-6	25
